# Deletion of Sphingosine Kinase 2 Attenuates Acute Kidney Injury in Mice with Hemolytic-Uremic Syndrome

**DOI:** 10.3390/ijms25147683

**Published:** 2024-07-12

**Authors:** Tina Müller, Nadine Krieg, Antonia I. Lange-Polovinkin, Bianka Wissuwa, Markus H. Gräler, Sophie Dennhardt, Sina M. Coldewey

**Affiliations:** 1 Department of Anesthesiology and Intensive Care Medicine, Jena University Hospital, 07743 Jena, Germany; tina.mueller2@med.uni-jena.de (T.M.); nadine.krieg@med.uni-jena.de (N.K.);; 2 ZIK Septomics Research Center, Jena University Hospital, 07743 Jena, Germany; 3 Center for Molecular Biomedicine (CMB) and Center for Sepsis Control and Care (CSCC), Jena University Hospital, 07743 Jena, Germany; 4 Center for Sepsis Control and Care (CSCC), Jena University Hospital, 07743 Jena, Germany

**Keywords:** hemolytic-uremic syndrome, Shiga toxin, sphingosine kinase, renal damage, kidney dysfunction, sphingolipids, sphingosine-1-phosphate, ceramides, cytokines

## Abstract

Typical hemolytic uremic syndrome (HUS) can occur as a severe systemic complication of infections with Shiga toxin (Stx)-producing *Escherichia coli*. Its pathology can be induced by Stx types, resulting in toxin-mediated damage to renal barriers, inflammation, and the development of acute kidney injury (AKI). Two sphingosine kinase (SphK) isozymes, SphK1 and SphK2, have been shown to be involved in barrier maintenance and renal inflammatory diseases. Therefore, we sought to determine their role in the pathogenesis of HUS. Experimental HUS was induced by the repeated administration of Stx2 in wild-type (WT) and SphK1 (SphK1^−/−^) or SphK2 (SphK2^−/−^) null mutant mice. Disease severity was evaluated by assessing clinical symptoms, renal injury and dysfunction, inflammatory status and sphingolipid levels on day 5 of HUS development. Renal inflammation and injury were found to be attenuated in the SphK2^−/−^ mice, but exacerbated in the SphK1^−/−^ mice compared to the WT mice. The divergent outcome appeared to be associated with oppositely altered sphingolipid levels. This study represents the first description of the distinct roles of SphK1^−/−^ and SphK2^−/−^ in the pathogenesis of HUS. The identification of sphingolipid metabolism as a potential target for HUS therapy represents a significant advance in the field of HUS research.

## 1. Introduction

Infection-associated hemolytic-uremic syndrome (HUS) is a rare but severe kidney disease, and the leading cause of acute kidney injury (AKI) in children worldwide [1]. A 2012–2023 literature review by Aldharman et al. found an annual incidence of 0.57–0.66 per 100,000 people [2]. However, children under the age of 10 und adults with increasing age are at greater risk [3,4,5,6]. Most cases of HUS are the result of a gastrointestinal infection with Shiga toxin (Stx)-producing *Escherichia coli* (STEC) (reviewed in [7]). STEC-associated HUS occurs sporadically or in outbreaks due to the consumption of contaminated food or water [8,9,10,11]. The typical clinical presentation of HUS is a prodromal gastroenteritis with occasionally bloody diarrhea, followed by a triad of non-immune microangiopathic anemia, thrombocytopenia, and AKI (reviewed in [12]). The characteristic damaged vascular endothelial bed in HUS is mainly caused by the Stx types, Stx1 and/or Stx2, which are the key virulence factors of STEC [13,14,15]. Stx1 and Stx2 share the same pathomechanism but differ in their toxic potency [16]. STEC can either produce Stx1 or Stx2, or both Stx types [17], regardless of the serotype [18]. Epidemiological data indicate that Stx2 is the predominant toxin associated with STEC-induced HUS [18,19]. The diagnosis of STEC infection relies on STEC serotype and Stx (sub-type) determination [20,21]. In low-income countries, the standard diagnostic tools for STEC infection diagnosis are not area-wide available due to missing infrastructure, the high costs of diagnostic tools, and difficulties in culturing STEC from patient samples [22]. Both toxin types enter the bloodstream via trans- and/or paracellular pathways (reviewed in [23,24]). The mediator of this pathological process is the glycosphingolipid globotriaosylceramide (Gb3) that is predominantly present in the endothelial cell membrane of the kidneys, brain, and gut ([25], reviewed in [26,27]). Gb3 acts as a receptor for Stx, which leads to the endocytosis of the Stx–Gb3 complex followed by a retrograde transport, which enables the enzymatically active moiety of Stx to inactivate the ribosomal protein synthesis of endothelial cells, causing ribosomal stress, cytokine production, and the apoptosis and necrosis of cells ([23,28], reviewed in [29]). In HUS development, this Stx-induced endothelial dysfunction is mainly apparent in kidney pathology [30]. The disruption of the endothelial barrier between blood vessels and tissue contributes to the development of vascular dysfunction [31], inflammation [32], recruitment of leukocytes [31,33], platelet thrombus formation [34,35], and thrombocytopenia [35]. Responsiveness to Stx has also been described in renal epithelial cells, supporting their role in contributing to kidney failure [36,37,38,39]. The common long-term consequences of HUS are renal interstitial fibrosis, impaired kidney function, proteinuria, hypertension, and neurological disorders [39,40,41]. Currently, there is no effective treatment to cure or prevent HUS development and its adverse outcomes.

The bioactive lipid sphingosine-1-phosphate (S1P) has drawn attention in medical research because of its multifaceted roles in physiology and pathophysiology, especially due to its regulating functions of endothelial (reviewed in [42]) and epithelial barrier maintenance [43,44], immune response (reviewed in [45]), and cell proliferation and survival (reviewed in [46]). The two isozymes of sphingosine kinase, sphingosine kinase (SphK) 1 and SphK2, catalyze S1P synthesis from the ceramide derivative sphingosine (Sph). Both enzymes are localized in distinct subcellular compartments (reviewed in [47]) and exhibit some redundancy in function (e.g., in embryonal development), but also distinct biochemical properties (e.g., substrate preference) (reviewed in [47,48]). Intracellularly synthesized S1P mainly mediates its multifaceted cellular functions via its five specific G-protein coupled receptors, namely S1P_1_ to S1P_5_, in an inside-out signaling fashion [49].

Null mutations of SphK1 (SphK1^−/−^) or SphK2 (SphK2^−/−^) in mice lead to altered S1P levels in the whole blood [50,51], plasma [50,51,52,53,54], and partially in tissues [50,53,54] in opposite ways, with SphK2^−/−^ leading to increased S1P levels. The modulation of S1P blood levels by deleting SphKs and/or altering S1P receptor signaling have been identified as valuable targets to improve the outcome of life-threatening infectious and inflammatory diseases leading to multiple organ failure, such as sepsis [53,55,56]. Experimental investigations on the role of SphKs in renal inflammation, a common consequence of sepsis, have yielded conflicting results. SphK2^−/−^ has been associated with the attenuation of kidney injury in murine models of cisplatin-induced kidney injury and unilateral ureteral obstruction [57,58], but aggravated kidney injury in a murine model of ischemia-reperfusion [59]. Alterations in renal damage severity in these models were mainly mediated by altered plasma S1P levels, an altered S1P receptor expression profile affecting vascular endothelial barrier permeability [59], or an attenuated inflammatory microenvironment [57,58]. SphK1^−/−^ had no effect on kidney injury severity in the ischemia-reperfusion model [59], but attenuated the long-term consequences of kidney injury, like, e.g., fibrosis [60].

The divergent effects of SphK1^−/−^ or SphK2^−/−^ on the maintenance of the vascular endothelial barrier and the severity of kidney injury during renal inflammation prompted us to investigate their roles in the development of HUS. We hypothesized that both kinases affect the early hallmarks of HUS, such as endothelial barrier damage and inflammatory response, thereby influencing the causal events of HUS development and the severity of HUS progression. We tested our hypothesis by comparing SphK1^−/−^ and SphK2^−/−^ mice with WT mice, employing a clinical relevant murine model of Stx2-induced experimental HUS [61]. We investigated clinical symptoms, renal injury and dysfunction, inflammatory status, and sphingolipid metabolism to examine the role of SphKs in HUS development.

## 2. Results

### 2.1. Deletion of SphK1 Aggravates HUS Disease Severity in Mice

**Figure 1:**
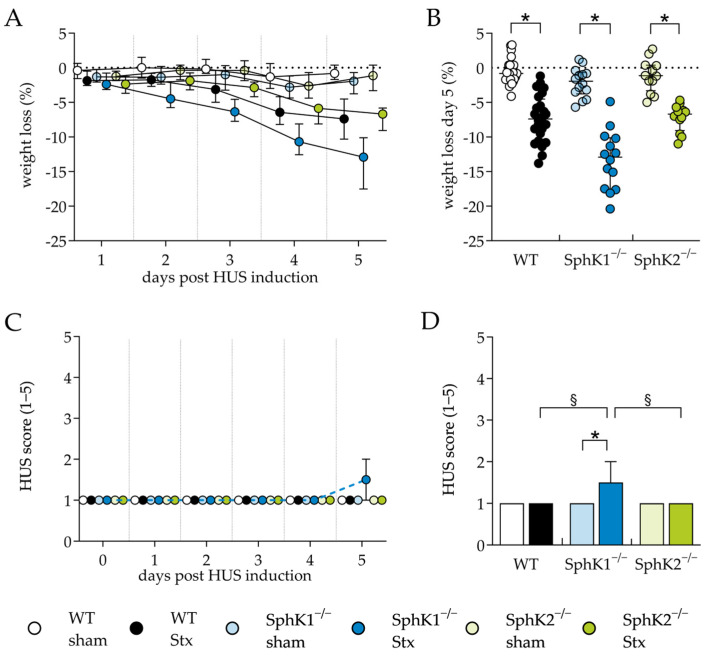
Weight loss and HUS score of WT, SphK1^−/−^, and SphK2^−/−^ mice with experimental hemolytic-uremic syndrome. Mice received vehicle (sham) or were challenged with Stx2 to induce experimental HUS and were followed up for 5 days (WT sham: *n* = 24, WT Stx: *n* = 24, SphK1^−/−^ sham: *n* = 14, SphK1^−/−^ Stx: *n* = 14, SphK2^−/−^ sham: *n* = 12, SphK2^−/−^ Stx: *n* = 12). (**A**) Progression of weight loss from day 1 to 5. (**B**) Overall weight loss on day 5. (**C**) HUS score from day 1 to 5. HUS score ranges from 1 = no signs of illness to 5 = dead. (**D**) HUS score on day 5. Data are expressed as (**A**,**C**) interleaved scatter plot, (**B**) scatter dot plot with median (interquartile range). and (**D**) bar plot with median (interquartile range) for *n* observations. Kruskal–Wallis test + Dunn’s multiple comparison test: * *p* < 0.05: Stx group vs. the corresponding sham group, § *p* < 0.05: Stx groups comparison. HUS, hemolytic-uremic syndrome; SphK, sphingosine kinase; Stx, Shiga toxin; WT, wild type.

The role of SphK deletion in HUS pathogenesis was investigated first by assessing the clinical presentation of mice with experimental HUS compared to sham mice. One of the hallmarks of HUS in mice is weight loss during the early stages of disease development [61,62]. Therefore, the weight loss of the mice was assessed over 5 days. A reduction in weight was observed in all Stx2-challenged mice compared to the corresponding sham mice during the experimental period (Figure 1A). On day 5, weight loss was the most pronounced in Stx2-challenged SphK1^−/−^ mice (12.9%), followed by the Stx2-challenged WT (7.4%) and SphK2^−/−^ mice (6.7%) (Figure 1B). Disease severity was further assessed by scoring the mice three times a day. The HUS score of the mice in all experimental groups remained unchanged until day 4 (Figure 1C). On day 5, the HUS score of the Stx2-challenged SphK1^−/−^ mice increased significantly compared to the corresponding sham mice, as well as the Stx2-challenged WT and SphK2^−/−^ mice (Figure 1D). Thus, the weight loss and score data indicate an exacerbation of disease severity in the SphK1^−/−^ mice, whereas SphK2 deletion did not affect the clinical presentation.

**Figure 2:**
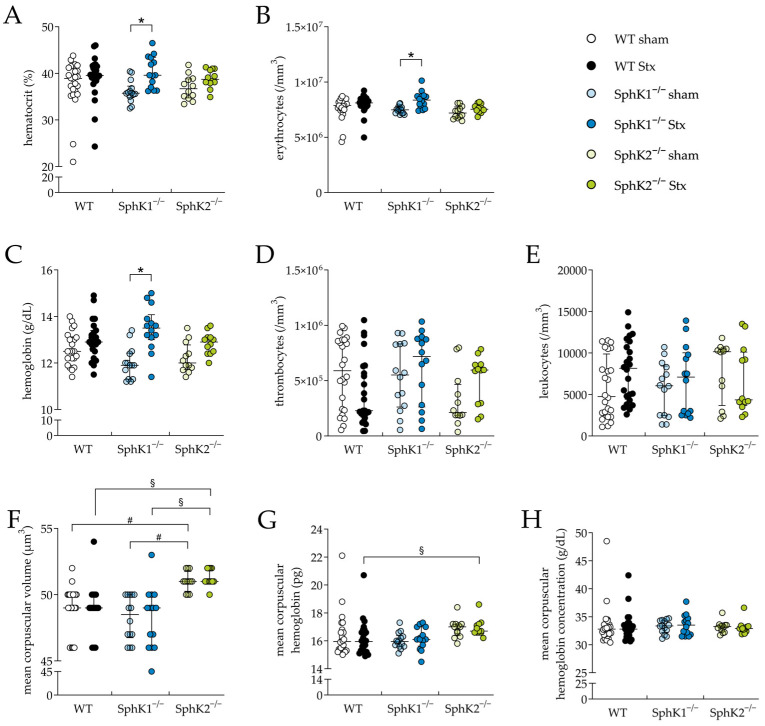
Hemogram analysis in WT, SphK1^−/−^, and SphK2^−/−^ mice with experimental hemolytic-uremic syndrome. Mice received vehicle (sham) or were challenged with Stx2 to induce experimental HUS. Whole-blood parameters were assessed by determining (**A**) hematocrit, (**B**) erythrocytes, (**C**) hemoglobin, (**D**) thrombocyte count, (**E**) leukocyte count, (**F**) mean corpuscular volume, (**G**) mean corpuscular hemoglobin, and (**H**) mean corpuscular hemoglobin concentration in whole blood drawn on day 5 (WT sham: *n* = 24, WT Stx: *n* = 24, SphK1^−/−^ sham: *n* = 14, SphK1^−/−^ Stx: *n* = 14, SphK2^−/−^ sham: *n* = 12, SphK2^−/−^ Stx: *n* = 12). Data are expressed as (**A**–**H**) scatter dot plot with median (interquartile range) for *n* observations. Kruskal–Wallis test + Dunn’s multiple comparison test: * *p* < 0.05: Stx group vs. the corresponding sham group, # *p* < 0.05: sham groups comparison, § *p* < 0.05: Stx groups comparison. SphK, sphingosine kinase; Stx, Shiga toxin; WT, wild type.

Whole-blood analysis was performed to further evaluate the HUS characteristics and severity, such as thrombocytopenia and hemoconcentration. In the Stx2-challenged SphK1^−/−^ mice, hematocrit (Figure 2A), erythrocyte count (Figure 2B) and hemoglobin concentration (Figure 2C) were significantly increased compared to the corresponding sham mice, indicating the development of hemoconcentration. In contrast, these blood parameters remained unchanged in the Stx2-challenged WT and SphK2^−/−^ mice compared to the corresponding sham mice. The count of thrombocytes (Figure 2D) and leukocytes (Figure 2E) were not significantly altered in any of the Stx2-challenged groups compared to the corresponding sham groups, suggesting no development of thrombocytopenia or lymphocytopenia in the mice during the experimental period, respectively. The erythrocyte index mean corpuscular volume (MCV, Figure 2F) was significantly increased in the sham and Stx2-challenged SphK2^−/−^ mice compared to the respective WT and SphK1^−/−^ mice. In addition, the erythrocyte index mean corpuscular hemoglobin (MCH, Figure 2G) was significantly increased in the Stx2-challenged SphK2^−/−^ mice compared to the Stx2-challenged WT mice. Alterations in both erythrocyte indices suggest a modulating effect on erythrocyte physiology by the deletion of SphK2. In contrary, the mean corpuscular hemoglobin concentration remained unchanged in all experimental groups (Figure 2H). In summary, the hematology data indicate an exacerbation of experimental HUS in the SphK1^−/−^ mice, but not in the SphK2^−/−^ mice. This is supported by the fact that hemograms of the Stx2-challenged SphK1^−/−^ mice on day 5 correspond to the hemograms of the Stx2-challenged WT mice on day 7 [61], when, as shown for the WT mice, disease development had already progressed further [61,63,64].

### 2.2. Deletion of SphK2 in Mice Attenuates Severe Kidney Injury and Dysfunction

**Figure 3:**
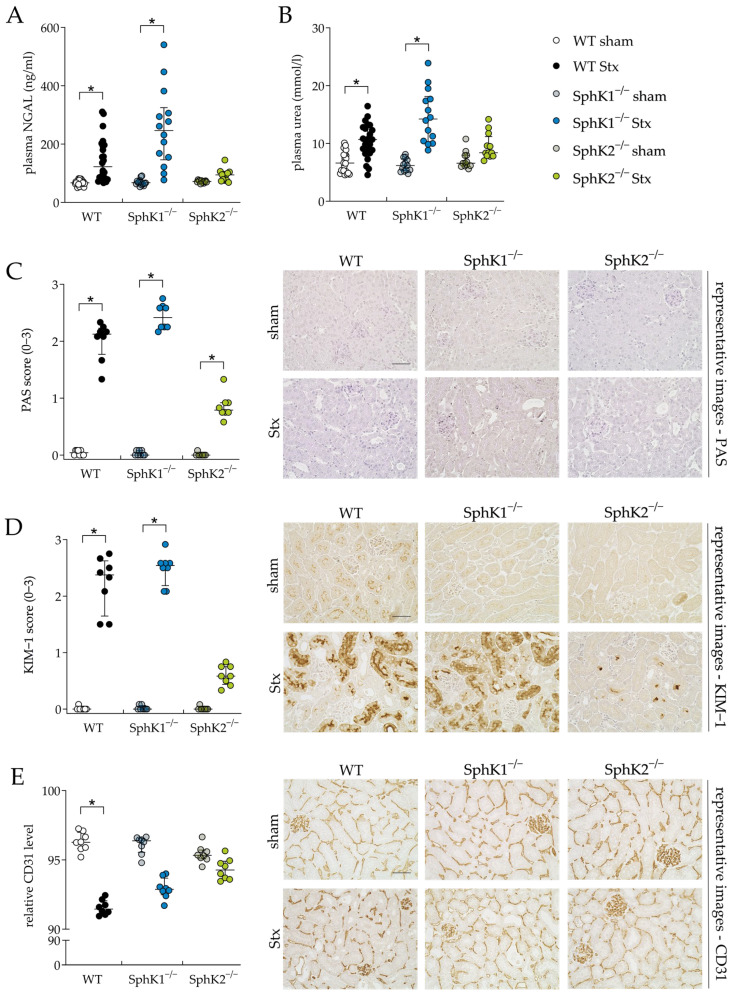
Kidney injury and dysfunction in WT, SphK1^−/−^, and SphK2^−/−^ mice with experimental hemolytic-uremic syndrome. Mice received vehicle (sham) or were challenged with Stx2 to induce experimental HUS. Kidney injury and dysfunction were assessed by determining plasma (**A**) NGAL (WT sham: *n* = 24, WT Stx: *n* = 24, SphK1^−/−^ sham: *n* = 14, SphK1^−/−^ Stx: *n* = 14, SphK2^−/−^ sham: *n* = 12, SphK2^−/−^ Stx: *n* = 12) and (**B**) urea (WT sham: *n* = 22, WT Stx: *n* = 23, SphK1^−/−^ sham: *n* = 13, SphK1^−/−^ Stx: *n* = 14, and SphK2^−/−^: *n* = 12) levels, respectively, on day 5. Kidney injury was further investigated by scoring of (**C**) PAS staining and immunohistological (**D**) KIM-1 and (**E**) CD31 staining of renal sections (*n* = 8 per group) on day 5. Bar = 50 µm (400× magnification). Data are expressed as (**A**–**E**) scatter dot plot with median (interquartile range) for *n* observations. Images (**C**–**E**) are representative for renal sections of 8 mice per group. Kruskal–Wallis test + Dunn’s multiple comparison test: * *p* < 0.05: Stx group vs. the corresponding sham group. CD31, cluster of differentiation 31; KIM-1, kidney injury molecule-1; NGAL, neutrophil gelatinase-associated lipocalin; PAS, periodic acid Schiff; SphK, sphingosine kinase; Stx, Shiga toxin; WT, wild type.

The kidney is the organ most affected by Stx in mice due to the high levels of Gb3 in renal vascular endothelial cells [26] and most likely also in tubular epithelial cells [65]. Therefore, we investigated the effect of either SphK1 or SphK2 deletion on renal damage and functional parameters during experimental HUS development. The Stx2-challenged WT and SphK1^−/−^ mice compared to the corresponding sham mice, showed significantly increased plasma levels of the renal damage biomarker neutrophil gelatinase-associated lipocalin (NGAL, Figure 3A) and renal dysfunction biomarker urea (Figure 3B). In the Stx2-challenged SphK2^−/−^ mice, compared to the corresponding sham mice, the levels of both markers remained unchanged. In line with these findings, periodic acid Schiff (PAS, Figure 3C) staining revealed significant pathological alterations in the kidneys of the Stx2-challenged WT and SphK1^−/−^ mice compared to the corresponding sham mice, respectively. In detail, histological staining revealed tubular dilatation, flattened tubular epithelium, PAS-positive protein casts, loose cellular material and severely damaged proximal tubules in the kidneys of these Stx2-challenged mice. Glomerular damage was not observed, as in murine HUS, tubular damage rather than glomerular damage occurs [62,66]. In the Stx2-challenged SphK2^−/−^ mice compared to the corresponding sham mice, the PAS score was also significantly increased. However, this increase was much lower compared to the Stx2-challenged WT and SphK1^−/−^ mice. The kidney injury molecule-1 (KIM-1) score was significantly increased in the Stx2-challenged WT and SphK1^−/−^ mice (Figure 3D), whereas the score of the Stx2-challenged SphK2^−/−^ mice was not significantly altered compared to the corresponding sham mice. Vascular endothelial cell damage was quantified by analyzing the relative levels of CD31, a histological marker for endothelial cell presence. The relative CD31 levels were significantly decreased in the renal tissue of the Stx2-challenged WT mice compared to the corresponding sham mice (Figure 3E). The decrease in relative CD31 levels was less pronounced in the Stx2-challenged SphK1^−/−^ and SphK2^−/−^ mice compared to the corresponding sham groups with almost equal relative CD31 levels in the Stx2-challenged SphK2^−/−^ mice compared to the corresponding sham group. Thus, the analysis of renal damage and dysfunction parameters indicated a protective effect of SphK2 deletion against the development of severe renal injury and dysfunction during the progression of experimental HUS.

### 2.3. Deletion of SphK2 Attenuates Immune Response during HUS Development

**Figure 4:**
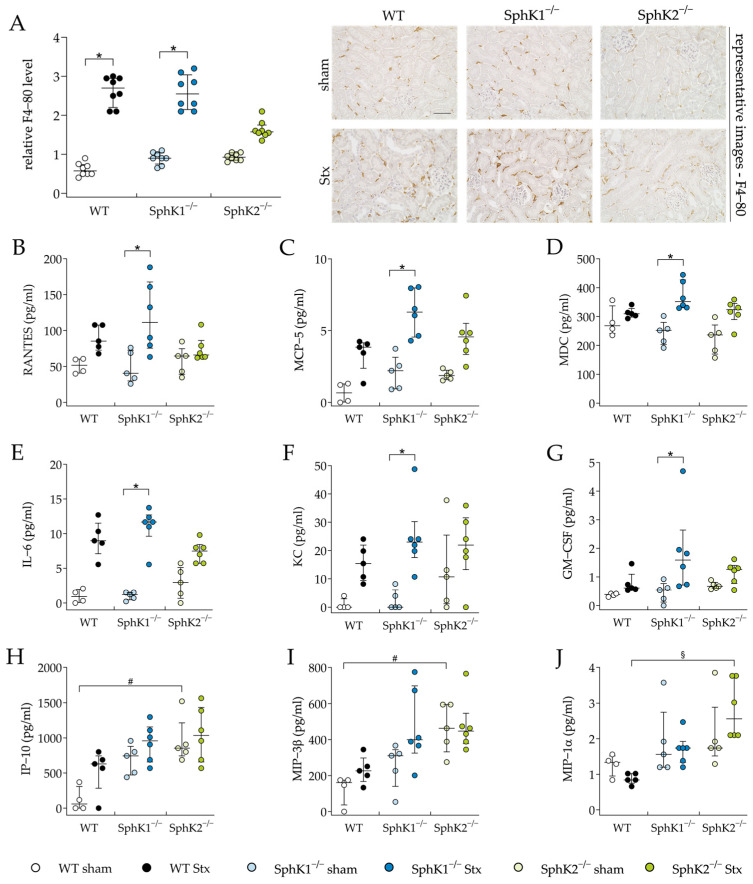
Macrophage invasion and cytokine release in WT, SphK1^−/−^, and SphK2^−/−^ mice with experimental hemolytic-uremic syndrome. Mice received vehicle (sham) or were challenged with Stx2 to induce experimental HUS. Macrophage invasion was assessed by determining the relative levels of (**A**) F4-80 in renal sections on day 5. Images are representative for renal sections of 8 mice per group. Bar = 50 µm (400× magnification). Cytokine levels of (**B**) RANTES, (**C**) MCP-5, (**D**) MDC, (**E**) IL-6, (**F**) KC, (**G**) GM-CSF, (**H**) IP-10, (**I**) MIP-3β, and (**J**) MIP-1α were measured on day 5 (WT sham: *n* = 4, WT Stx: *n* = 5, SphK1^−/−^ sham: *n* = 5, SphK1^−/−^ Stx: *n* = 6, SphK2^−/−^ sham: *n* = 5, SphK2^−/−^ Stx: *n* = 6). Data are expressed as (**A**–**J**) scatter dot plot with median (interquartile range) for *n* observations. Kruskal–Wallis test + Dunn’s multiple comparison test: * *p* < 0.05: Stx group vs. the corresponding sham group, # *p* < 0.05: sham groups comparison, § *p* < 0.05: Stx groups comparison. GM-CSF, granulocyte-macrophage colony-stimulating factor; IL-6, interleukin-6; KC, keratinocyte-derived chemokine; MCP-5, monocyte chemoattractant protein-5; MDC, macrophage-derived chemokine; RANTES, Regulated And Normal T cell Expressed and Secreted; IP-10, interferon gamma-induced protein 10; MIP-3β, macrophage inflammatory protein-3-beta; MIP1α, macrophage inflammatory protein-3-alpha; SphK, sphingosine kinase; Stx, Shiga toxin; WT, wild type.

Kidney injury and dysfunction during HUS development can be aggravated by the renal infiltration of immune cells that were initially attracted by cytokines secreted by the Stx-damaged endothelial and epithelial cells (reviewed in [67]). Therefore, we exemplarily investigated renal immune cell infiltration by quantifying the relative F4-80 levels representing macrophage invasion, and examined cytokine secretion by a plasma cytokine and chemokine analysis. The relative F4-80 levels were significantly elevated in the Stx2-challenged WT and SphK1^−/−^ mice compared to the corresponding sham groups, but not in the Stx2-challenged SphK2^−/−^ mice (Figure 4A). In the Stx2-challenged SphK1^−/−^ mice compared to the corresponding sham mice, the plasma levels of the cytokines interleukin (IL)-6 and granulocyte-macrophage colony-stimulating factor (GM-CSF) and the chemokines Regulated And Normal T cell Expressed and Secreted (RANTES), monocyte chemoattractant protein (MCP)-5, macrophage-derived chemokine (MDC), and keratinocyte-derived chemokine (KC) were significantly increased (Figure 4B–G). In the SphK2^−/−^ sham mice compared to the WT sham mice, the plasma levels of the chemokines interferon gamma-induced protein (IP)-10 (Figure 4H) and macrophage inflammatory protein-3-beta (MIP-3β) (Figure 4I) were significantly increased. Further, in the Stx2-challenged SphK2^−/−^ mice compared to the Stx2-challenged WT mice, significantly increased plasma levels of macrophage inflammatory protein-1-alpha (MIP-1α) were observed (Figure 4J). The levels of other cytokines and chemokines included in the plasma analysis remained unchanged in all experimental groups (Supplementary Table S7). In summary, the deletion of SphK1 aggravated the inflammatory microenvironment in mice with experimental HUS. The SphK2^−/−^ mice exhibited alterations in their intrinsic chemokine balance and were protected from renal macrophage invasion during the development of experimental HUS.

### 2.4. Deletion of SphK Alters Sphingolipid Profile of Plasma and Renal Tissue

**Figure 5:**
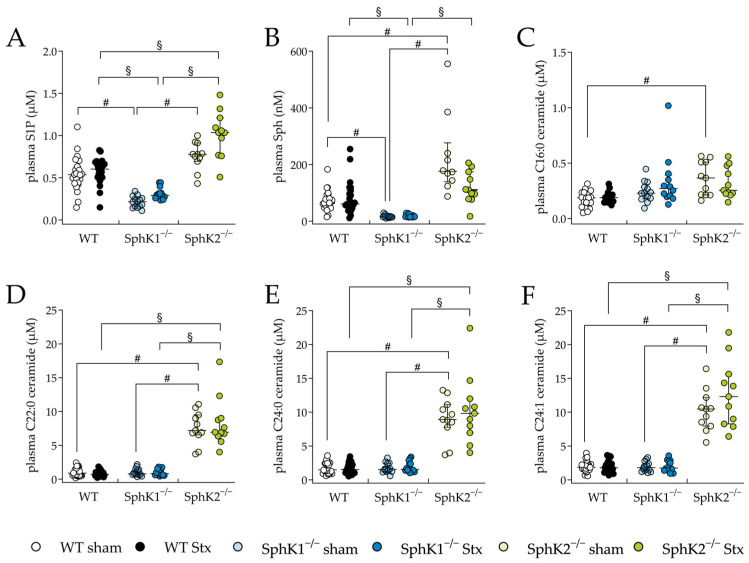
Sphingolipid levels in plasma from WT, SphK1^−/−^, and SphK2^−/−^ mice with experimental hemolytic-uremic syndrome. Mice received vehicle (sham) or were challenged with Stx2 to induce experimental HUS. To assess changes in plasma sphingolipid levels from mice with experimental HUS, levels of (**A**) S1P, (**B**) Sph and the ceramide (Cer) species (**C**) C16:0 Cer, (**D**) C22:0 Cer, (**E**) C24:0 Cer, and (**F**) C24:1 Cer were determined by LC-MS/MS. Data are expressed as scatter dot plots with median (interquartile range) for *n* observations. (**A**,**D**–**F**): WT: *n* = 24, SphK1^−/−^: *n* = 14, SphK2^−/−^: *n* = 11, (**B**): WT sham: *n* = 24, WT Stx: *n* = 23, SphK1^−/−^ sham: *n* = 13, SphK1^−/−^ Stx: *n* = 13, SphK2^−/−^ sham: *n* = 10, SphK2^−/−^ Stx: *n* = 10, (**C**): WT sham: *n* = 14, WT Stx: *n* = 11, SphK1^−/−^ sham: *n* = 13, SphK1^−/−^ Stx: *n* = 12, SphK2^−/−^ sham: *n* = 10, SphK2^−/−^ Stx: *n* = 10. Kruskal–Wallis test + Dunn’s multiple comparison test: # *p* < 0.05: sham groups comparison, § *p* < 0.05: Stx groups comparison. Cer, ceramide; HUS, hemolytic-uremic syndrome; S1P, sphingosine-1-phosphate; Sph, sphingosine; SphK, sphingosine kinase; Stx, Shiga toxin; WT, wild type.

The plasma and renal sphingolipid levels were analyzed to investigate changes resulting from the deletion of SphKs, as well as from possible changes during HUS development. In the SphK1^−/−^ sham mice and Stx2-challenged SphK1^−/−^ mice, the plasma S1P (Figure 5A) and Sph (Figure 5B) levels were significantly decreased compared to the WT sham mice or Stx2-challenged WT mice, respectively. In the SphK2^−/−^ sham mice, the plasma S1P levels were increased compared to the WT sham mice (statistically non-significant) and the Sph levels were significantly increased compared to the WT sham mice. In the Stx2-challenged SphK2^−/−^ mice, the plasma S1P levels were significantly increased compared to the Stx2-challenged WT mice, whereas the Sph levels compared to the Stx2-challengend WT mice remained unchanged. A comparison of the SphK1^−/−^ with SphK2^−/−^ sham mice and comparison of the Stx2-challenged SphK1^−/−^ with Stx2-challenged SphK2^−/−^ mice revealed significantly altered S1P and Sph levels. In the SphK2^−/−^ sham mice compared to the WT sham mice, the plasma levels of C16:0 ceramide were significantly increased (Figure 5C). Interestingly, in both the SphK2^−/−^ sham and Stx2-challenged SphK2^−/−^ mice, the levels of some ceramide species (Figure 5D–F) were significantly increased compared to the respective WT and SphK1^−/−^ mice groups. Furthermore, comparisons of the Stx2-challenged mice with the corresponding sham groups showed no statistically significant changes in the levels of S1P, Sph, or ceramide species (Figure 5A–F).

**Figure 6:**
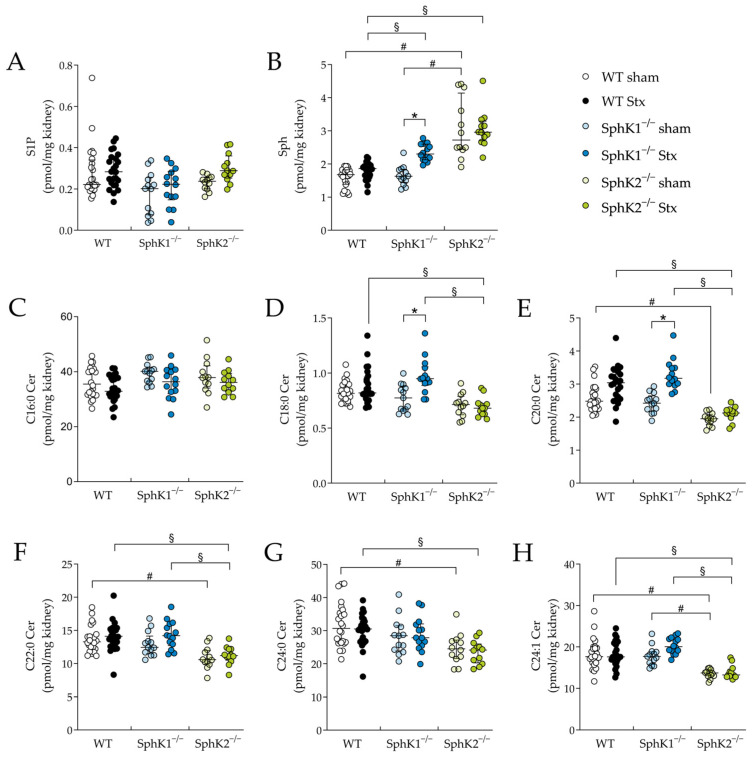
Sphingolipid levels in renal tissue from WT, SphK1^−/−^ and SphK2^−/−^ mice with experimental hemolytic-uremic syndrome. Mice received vehicle (sham) or were challenged with Stx2 to induce experimental HUS. To assess changes in sphingolipid levels in renal tissue from mice with experimental HUS, levels of (**A**) S1P, (**B**) Sph and ceramide species (**C**) C16:0 Cer, (**D**) C18:0 Cer, (**E**) C20:0 Cer, (**F**) C22:0 Cer, (**G**) C24:0 Cer, and (**H**) C24:1 Cer were determined by LC-MS/MS. Data are expressed as scatter dot plots with median (interquartile range) for *n* observations. (**A**–**H**): WT sham: *n* = 24, WT Stx: *n* = 24, SphK1^−/−^ sham: *n* = 13–14, SphK1^−/−^ Stx: *n* = 14, SphK2^−/−^ sham: *n* = 12, SphK2^−/−^ Stx: *n* = 12. Kruskal–Wallis test + Dunn’s multiple comparison test: * *p* < 0.05: Stx group vs. the corresponding sham group, # *p* < 0.05: sham groups comparison, § *p* < 0.05: Stx groups comparison. Cer, ceramide; HUS, hemolytic-uremic syndrome; S1P, sphingosine-1-phosphate; Sph, sphingosine; Stx, Shiga toxin; SphK, sphingosine kinase; WT, wild type.

In renal tissue, the S1P levels remained unchanged in all Stx2-challenged mice compared to the corresponding sham groups (Figure 6A). In the SphK2^−/−^ sham mice compared to the WT and SphK1^−/−^ sham mice, the renal Sph levels were significantly increased (Figure 6B). Furthermore, in the Stx2-challenged SphK1^−/−^ and SphK2^−/−^ mice compared to the Stx2-challenged WT mice, the renal Sph levels were significantly increased. In the Stx2-challenged SphK1^−/−^ mice compared to the corresponding sham mice, the renal Sph levels were significantly increased. In all experimental groups, the levels of C16:0 ceramide in the renal tissue remained unchanged (Figure 6C). In the renal tissue of the SphK2^−/−^ sham mice compared to the WT sham mice and of the Stx2-challenged SphK2^−/−^ mice compared to the Stx2-challenged WT mice, the levels of C20:0 (Figure 6E), C22:0 (Figure 6F), C24:0 (Figure 6G), and C24:1 (Figure 6H) ceramide species were significantly decreased. Furthermore, in the renal tissue of the Stx2-challenged SphK2^−/−^ mice compared to the Stx2-challenged SphK1^−/−^ mice, the levels of C18:0 (Figure 6D), C20:0 (Figure 6E), C22:0 (Figure 6F), and C24:1 (Figure 6H) ceramide species were significantly decreased. Finally, in the Stx2-challenged SphK1^−/−^ mice compared to the corresponding sham mice, a significant increase in renal C18:0 and C20:0 ceramide levels was observed.

Summarizing the sphingolipid profile analyses, the SphK2^−/−^ mice compared to the WT and SphK1^−/−^ mice showed increased plasma levels of S1P, Sph, and ceramide species and increased renal Sph, but decreased renal ceramide levels. The SphK1^−/−^ mice compared to the WT mice showed decreased plasma S1P levels, but the Stx2-challenge increased renal Sph and partially the ceramide levels in the SphK1^−/−^ mice. Renal S1P levels were not affected by the deletion of SphK1 or SphK2.

## 3. Discussion

To date, a causal therapy counteracting HUS development and thereby preventing the severe long-term sequelae of this orphan systemic disease is still missing. We suggested that both SphKs influence the early hallmarks of HUS development, namely, damage of the renal barriers and the immune response, events paving the way for the HUS defining triad of thrombocytopenia, hemolytic anemia, and acute kidney injury.

In the present study, we investigated, for the first time, the role of SphKs during HUS development and elucidated that the deletion of SphK1 or SphK2 both do affect HUS pathogenesis in an opposing manner. The deletion of SphK1 in mice exacerbated the disease severity of experimental HUS, as evidenced primarily by a worsening of clinical presentation, and increased plasma cytokine and chemokine levels. Both SphK1^−/−^ mice groups differed phenotypically, mainly in their reduced plasma S1P and Sph levels compared to the WT and SphK2^−/−^ mice. In contrary, SphK2 deletion attenuated HUS disease severity, as evidenced primarily by the prevention of severe proximal tubule damage, the renal infiltration of macrophages, and kidney dysfunction. Phenotypically, the main differences in both SphK2^−/−^ mice groups compared to the WT and SphK1^−/−^ mice were their increased plasma S1P, Sph, and ceramide levels, but decreased renal ceramide levels and alterations in their chemokine balance. Interestingly, the induction of experimental HUS did not influence the plasma S1P levels in the WT or any of the SphK null mutant mice. In other inflammatory diseases such as sepsis, plasma or serum S1P levels are known to severely drop in humans [55,68], WT mice [53,56], and SphK1^−/−^ mice [53], but not in SphK2^−/−^ mice [53,55]. However, we only determined the plasma S1P levels on day 5 post-induction of experimental HUS. The levels might have changed at earlier points in time or even at later time points when HUS disease progression reached its maximum.

Investigations of the development of thrombocytopenia based on blood cell analysis contradicted the development of this part of the HUS triad in WT, SphK1^−/−^, and SphK2^−/−^ mice with experimental HUS in this study at first glance. We already previously described mice in our HUS model to develop only weak thrombocytopenia that might be masked by hypovolemia and consecutive hemoconcentration [61,63], as was also observed in the SphK1^−/−^ mice with experimental HUS in the present study. However, blood cell analysis further revealed significantly increased erythrocyte indices, like the mean corpuscular volume in both SphK2^−/−^ mice groups compared to the WT and SphK1^−/−^ mice groups, indicating an intrinsic effect on erythrocyte physiology. Erythrocytes from SphK2^−/−^ mice are known to accumulate S1P [69], and erythrocyte S1P accumulation was shown to result in the enlargement of erythrocytes, as well as an altered turnover rate [70]. Furthermore, elevated erythrocyte S1P concentration protects against tissue hypoxia [71], a critical part of AKI pathology [72]. Therefore, this phenotypical trait of SphK2^−/−^ mice might also be beneficial for attenuating AKI pathology during HUS development.

The opposing effects of the deletion of SphK1 or SphK2 in mice on kidney injury and dysfunction can be presumably attributed partly to their distinct plasma and renal sphingolipid profiles. Differences between both genotypes in the Stx2-induced damage of renal barriers appeared in the proximal tubule epithelia (Figure 3D), but not in the vascular endothelium (Figure 3E). A loss of endothelial cells was neither induced in the SphK1^−/−^ nor in the SphK2^−/−^ mice upon the induction of experimental HUS, but it was in the WT mice with experimental HUS. On the one hand, circulating S1P enhances vascular endothelial barriers under inflammatory conditions in vivo [73,74], and SphK1^−/−^ and SphK2^−/−^ mice have distinct plasma S1P levels, with SphK2^−/−^ mice containing increased levels of plasma S1P [50,52,53,75], suggesting the SphK2^−/−^ phenotype to prevent mice from vascular endothelial barrier damage. On the other hand, there is controversy about SphK1 [76,77] and SphK2 [53,78] deletion in mice and their roles in vascular barrier maintenance during inflammation. In our model, both genotypes of SphK^−/−^ mice were prevented from suffering a severe loss of endothelial cells. This supports an enhancement of the vascular endothelium by SphK deletion that is not accountable to circulating S1P levels alone. The discrepancy with other models showing decreased vascular endothelial barrier permeability in SphK1^−/−^ or SphK2^−/−^ mice might result from the different analytical methods used to investigate endothelial barrier damage [53,76,77]. While we analyzed the presence of endothelial cells by the quantification of relative CD31 levels, the above-cited studies investigated vascular permeability in functionality assays.

However, damage of the proximal tubule epithelia was only prevented in the SphK2^−/−^ mice with experimental HUS, and their sphingolipid profile in the kidneys might be the key to this difference. The SphK2^−/−^ mice with experimental HUS contained increased S1P, Sph, and ceramide species levels in their plasma compared to the WT and SphK1^−/−^ mice upon the induction of experimental HUS. In a human study on systemic lupus erythematosus, this profile of plasma sphingolipids was suggested to indicate renal impairment [79]. However, the renal tissue of the SphK2^−/−^ mice with experimental HUS contained reduced levels of long-chain (C16 to C22) and very-long-chain (C24) ceramide species in comparison to the WT and SphK1^−/−^ mice upon the induction of experimental HUS. In contrary, the C18:0 and C20:0 ceramide levels were only increased by the induction of experimental HUS in the SphK1^−/−^ mice. In different murine models of AKI, the generation of long-chain and very-long-chain ceramides was suggested to serve cells as a response to acute renal stress, and the cytotoxic ceramide species involved in AKI are most likely long-chain or very-long-chain ceramide species (reviewed in [80]). The inhibition of ceramide synthesis attenuated the induction of proximal tubule cell death by various stimuli in different in vitro models [81,82,83]. Therefore, decreased levels of long-chain and very-long-chain ceramide species in the kidneys of the SphK2^−/−^ mice with experimental HUS compared to the WT and SphK1^−/−^ mice with experimental HUS might have protected them from the induction of severe damage of proximal tubule epithelial cells, whereas in the SphK1^−/−^ mice with experimental HUS, increased long-chain ceramide levels might have contributed to enhanced proximal tubule epithelial damage. Nonetheless, several studies have provided evidence that the modulation of the SphK–S1P receptor axis influences the damage of proximal tubular cells, as well as the induction of AKI in mice (reviewed in [84]), leading to the assumptions that (a) renal ceramide levels did not solely account for the severity of proximal tubular damage and kidney injury severity and (b) SphK mutant mice might have altered renal S1P receptor expression profiles. The S1P receptor expression in SphK mutant mice is rarely investigated. To our knowledge, only Eresch et al. explored the S1P receptor expression in the retinae of SphK2^−/−^ mice, showing alterations in the expression of S1P receptor type 1 and 4 [85]. Since murine models of HUS characteristically show damage primarily to the tubules [61,62,66], we are limited to a statement about the influence of SphKs on tubular damage. No statement can be made about the influence on glomerular damage that is characteristically induced in addition to tubular damage in human HUS patients [86,87]. Furthermore, as we chose the endpoint of the experimental investigation on day 5, when HUS development was shown to not have reached its maximum [61,64], we cannot exclude that HUS development in SphK2^−/−^ mice is delayed and might occur at a later point in time.

The prevention of severe proximal tubular damage might explain the reduced macrophage infiltration of renal tissue in the SphK2^−/−^ mice with experimental HUS. Human proximal tubule epithelial cells secrete different cytokines, like TNF-α, IL-1β, IL-6, and IL-8 [36,88], in response to Stx-induced damage and might, thus, attract immune cells that further infiltrate damaged tissues, potentiating renal damage. In mice, the toxin alpha-hemolysin from uropathogenic *E. coli* upregulated GM-CSF secretion by renal epithelial cells, resulting in M1 macrophage accumulation in the kidneys and the induction of AKI [89]. Therefore, in the SphK1^−/−^ mice with experimental HUS, the enhanced damage of proximal tubule epithelial cells might have resulted in the detected increased levels of cytokines and chemokines in our study. In contrary, the SphK2^−/−^ mice showed alterations in their intrinsic chemokine ligand profiles compared to the WT mice in our study. The levels of macrophage inflammatory protein (MIP)-3β, a ligand of CCR7, and interferon γ (IFN-γ) and inducible protein (IP)-10 levels were intrinsically significantly increased in the SphK2^−/−^ sham mice. Furthermore, the SphK2^−/−^ sham mice contained elevated plasma IFN-γ levels, even though they did not statistically significantly increase compared to the WT sham mice. This chemokine profile suggests an alternated T cell profile, because increased levels of MIP-3β are known to support CD4^+^ and CD8^+^ T cell egress from lymph nodes and stimulated T cells’ secretion of IFN-γ (reviewed in [90]). This phenotype is in accordance with SphK2^−/−^ mice in a model of folic acid (FA)-induced kidney fibrosis. The kidneys of these SphK2^−/−^ mice have been described to exhibit a greater expression of IFN-γ and IFN-γ-responsive genes, like, e.g., IP-10, compared to WT or SphK1^−/−^ mice. Furthermore, the splenic T cells from SphK2^−/−^ sham mice were hyperproliferative [91,92] and produced more IFN-γ than WT or SphK1^−/−^ mice [53,91,92]. Increased IFN-γ levels attenuated FA-induced kidney fibrosis in mice and protected them from severe kidney dysfunction [91]. These results were investigated in a long-term model of 14 days [91] and suggest the deletion of SphK2 to also prevent long-term sequelae of HUS. This presumption is further supported by the fact that SphK2 deletion also protected the mice from kidney fibrosis induced by unilateral ureteral obstruction [54]. On the contrary, the Stx2 challenge significantly elevated MIP-1α in the SphK2^−/−^ mice compared to the WT mice with HUS. MIP-1α levels have been shown to significantly increase in in vitro [36] and in vivo [93] models of Stx2 or Stx2/LPS-induced experimental HUS. The elevated concentration of MIP-1α in the plasma of the SphK2^−/−^ mice with experimental HUS contradicts the results of the decreased macrophage infiltration of the renal tissue of the SphK2^−/−^ mice. The unchanged plasma levels of other chemoattractants, such as RANTES, one main trigger of macrophage recruitment to the kidney during HUS development [93], might explain these differences. On the other hand, the elevation of plasma MIP-1α might also indicate a delay in macrophage infiltration in SphK2^−/−^ mice with experimental HUS, and, thus, in the general HUS pathogenesis. The significantly increased PAS scores of the SphK2^−/−^ mice with experimental HUS support this presumption.

In conclusion, we elucidated, for the first time, the impact of the SphKs in the pathogenesis of HUS. The study provides initial evidence to support the hypothesis that SphK1 and SphK2 play distinct roles in the pathogenesis of HUS. Renal inflammation and injury were attenuated in the SphK2^−/−^ mice but exacerbated in the SphK1^−/−^ mice compared to the WT mice. Since the different results observed in the two mutants were related to oppositely altered sphingolipid levels in the kidneys, these effects could be mediated—at least in part—by altered sphingolipid metabolism. Further investigations are required to validate and substantiate our results. However, the identification of the sphingolipid metabolism as a potential target for HUS therapy represents a significant advance in the field of HUS research.

## 4. Materials and Methods

### 4.1. Compounds

Ringer lactate solution *ad us. vet.* (Wirtschaftsgenossenschaft deutscher Tierärzte (WDT), Garbsen, Germany), NaCl solution 0.9% *ad us. vet.* (WDT), ketamine (Ketabel 100 mg/mL, bela pharm, Vechta, Germany), xylazine (Rompun^®^ 2% *ad us. vet.*, Elanco, Bad Homburg, Germany), heparin-natrium (PZN 09929393, Braun SE, Melsungen, Germany), 5% buffered formaldehyde solution (27261, Fishar, Saarbrücken, Germany), ethanol (11094.01, Carl Roth, Karlsruhe, Germany), xylene (9713.5, Carl Roth), paraffin (12617956/6774060, Shandon Dignostics, Runcorn, UK), 30% H_2_O_2_ (8070.1, Carl Roth), target retrieval solution (S1699, Agilent Dako, Waldbronn, Germany), milk powder (T145.2, Carl Roth), tris(hydroxymethyl)aminomethane (TRIS, 4855.5, Carl Roth), bovine serum albumin (11930.04, Serva, Heidelberg, Germany), rabbit serum (P30-1100, PAN-Biotech, Aidenbach, Germany), hemalaun (T865.2, Carl Roth), Tween^®^ 20 (P9416, Sigma Aldrich, Taufkirchen, Germany), methanol (349662500, Diagonal, Münster, Germany), formic acid (5355.1, Carl Roth), acetonitrile (93402500, Diagonal), 15:0 ceramide (2037, Biotrend, Köln, Germany), d17:1 S1P (860641P-1MG, Sigma Aldrich), and d17:1 sphingosine (860641P-1MG, Sigma Aldrich) were used.

### 4.2. Characterization of Stx2

There is evidence that Stx2 is more frequently associated with the development of HUS than other types [18,19]. Thus, Stx2 was purified in our laboratory using fast protein liquid chromatography from an O157:H7 EHEC strain 86–24 patient isolate (described in detail in [61]). The strain exclusively produces Stx2 [94,95]. The cytotoxicity and LD_50_ of the purified Stx2 were determined with Vero cells using a neutral red assay (TOX4, Sigma Aldrich). The LD_50_ of the purified Stx2 was 9.51 pg/mL.

### 4.3. Study Design und Animal Experiments

The experimental induction of HUS was achieved through the repeated administration of Stx2 to WT mice and mutant mice with disrupted SphK1 (SphK1^−/−^) or SphK2 (SphK2^−/−^) alleles, as previously described [96,97]. The SphK1^−/−^ and SphK2^−/−^ mice were kindly provided by Richard L. Proia (NIH, Bethesda, MD, USA). The concentrations of Stx2 employed and the selected injection regimen were based on our previous investigations and findings [64]. Given that HUS is characterized by the occurrence of AKI, regardless of the initiating agent, the employed model was well-suited to investigate the impact of novel pharmacological strategies on AKI, irrespective of the toxin type.

The mice used in this study were bred in a specific pathogen-free environment. Throughout the acclimatization (7 d) and experimental period (5 d), the ambient temperature was 21 °C ± 2 °C and the relative humidity was 55% ± 10%. A maximum of five mice per cage were housed under standardized laboratory conditions and with standard rodent chow and water ad libitum at the animal facility of the university hospital of Jena. To determine the required sample size per group a priori, the software G*Power 3.1.9.7 was used, as described by Faul et al., 2007 [98]. Efforts were made to keep the number of animals as low as possible. Experimental HUS was induced in 10- to 17-week-old male C57BL/6J WT, SphK1^−/−^, and SphK2^−/−^ mice by i.v. injections of Stx2 from one purification batch on days 0 and 3, accompanied by volume resuscitation with 800 µL of Ringer lactate solution s.c. 3 times daily. Mice weighting 20–30 g received 2x 25 ng Stx2/kg bodyweight (WT Stx (*n* = 24), SphK1^−/−^ Stx (*n =* 14), SphK2^−/−^ Stx (*n* = 12)), or 0.9% NaCl (WT sham (*n* = 24), SphK1^−/−^ sham (*n* = 14), and SphK2^−/−^ sham (*n* = 12)). The WT and mutant mice were held in separate cages. Where possible, both treatment groups were distributed evenly in a cage. If there was an odd number of animals per cage, the last animal was distributed at random. Mice and treatment identification was ensured by pollutant-free color-coded tail markings. Bodyweight and HUS score (Supplementary Table S1) were monitored for 5 days, as described previously [61]. Mice were sacrificed on day 5 or upon reaching humane endpoints (see termination criteria in Supplementary Table S1) to comply with ethical regulations. The animals were exsanguinated in deep ketamine/xylazine anesthesia (100 mg/kg bodyweight ketamine; 10 mg/kg bodyweight xylazine). All in vivo experiments were approved by the regional animal welfare committee (Thuringian State Office for Food Safety and Consumer Protection, Bad Langensalza, Germany, registration number 02-073/16) and were performed in accordance with German legislation and the approved ARRIVE guidelines. Blood withdrawal occurred from the vena cava, followed by whole-animal perfusion with 0.9% NaCl to remove the remaining blood cells, and organ harvesting. Assessments of the outcome measures for the mice were carried out in a blinded manner and allocations were conducted before statistical analysis.

### 4.4. Blood Analysis

Hemograms from natrium heparin anti-coagulated blood were compiled using the hematology device scil Vet abc Plus^+^ (scil animal care company GmbH, Viernheim, Germany). Plasma was obtained by centrifuging blood for 10 min at 3000× *g* and 4 °C. The plasma levels of urea and NGAL were analyzed with commercial kits according to the manufacturers’ instructions (Supplementary Table S2). Values outside the interpolatable range were excluded from the analysis. Cytokines and chemokines were determined in the plasma by Bio-Plex Pro Mouse Chemokine 31-plex panel assay (Bio-Rad, Feldkirchen, Germany). The cytokines IL-1β, −2, −4, −6, −10, and −16, IFN-γ, TNF-α, and GM-CSF and the chemokines CCL1, −2, −3, −4, −5, −7, −11, −12, −17, −19, −20, −22, −24, and −27, CX3CL1, and CXCL1, −5, −10, −11, −12, −13, and −16 were measured according to the manufacturer’s instructions. The assays were performed in one batch, with samples randomly distributed. Data were collected and analyzed using a Bio-Plex^®^ 200 instrument equipped with the Bio-Plex Manager 6.1.1 software (Bio-Rad). Values below the interpolatable range were assumed to approach being near 0 and, consequently, those values were set to 0 for statistics.

### 4.5. Tissue Preparation, Histopathology, and Immunohistochemistry

Generally, the kidneys were fixed with 5% buffered formaldehyde solution for at least 72 h at 4 °C. The dehydration of the kidneys occurred through a rising ethanol series, followed by clearing treatment with xylene, and subsequently, they were embedded in paraffin blocks. Renal sections (2 µm) were deparaffinized and rehydrated as described previously [61]. Histomorphological changes were determined with a PAS staining kit, which was performed according to the manufacturer’s instructions (Supplementary Table S2). Staining of kidney injury molecule-1 (KIM-1), F4-80, and CD31 was used for immunohistochemical evaluation. First, endogenous peroxidase activity was blocked in 3% H_2_O_2_ for 10–20 min at room temperature and antigen retrieval was performed with target retrieval solution for 10–15 min at 110 °C using a pressure cooker (Biocare Medical, Pacheco, CA, USA). To block unspecific binding sites, sections were treated with milk powder or serum (Supplementary Table S2), as well as applying an avidin/biotin blocking kit (15 min/L min; Supplementary Table S2). Further, sections were incubated with primary antibody overnight at 4 °C and incubated with secondary antibody for 30 min at room temperature (Supplementary Table S3). Horseradish Peroxidase (HRP) conjugation was performed according to the instructions of the VectaStain Elite ABC Kit and the ImmPACT DAB Peroxidase HRP Substrate Kit (Supplementary Table S2). During the immunohistochemical staining, steps sections were washed with TRIS buffer: 50 mM TRIS, 300 mM NaCl, pH was adjusted to 7.6 with HCl, 0.04% Tween^®^ 20. After counterstaining with hemalaun, sections were dehydrated by a rising ethanol series and xylene and mounted for observation. Representative images were captured after performing white balance and auto exposure at a magnification of 400× using a KEYENCE BZ-X800 microscope and the software BZ-X800 viewer Ver. 1.1.1 (Keyence, Neu-Isenburg, Germany). Each representative image was also stored as single, high-resolution file in the supplementary zip archive (Supplementary Material Microscopy Images File S1).

### 4.6. Quantification of Histopathological and Immunohistochemical Staining

PAS: For the quantification of tubular injury, 12 cortical fields per animal were randomly graded for signs of tubular injury (i.e., brush border loss, epithelial cell flattening, vacuolization) using a scoring system from 0 to 3: 0: no damage, 1: max. 25%, 2: 25–50%, and 3: >50%.

KIM-1: In total, 12 cortical fields per animal were evaluated by using a scoring system from 0 to 3: 0: <25%, 1: 25–50%, 3: 50–75%, and 3: >75% strong positive staining per visual field (brown).

F4-80: Renal macrophage invasion was quantified by counting intersections with overlapping positive brown staining in 20 adjacent cortical grid areas.

CD31: Renal endothelial barrier damage was quantified by counting caskets with positive brown staining in 20 adjacent cortical grid areas.

For the analysis of histopathological and immunohistochemical staining, quantification was performed blinded at a magnification of 400×. The mean value of all cortical fields or grid areas (10 × 10 caskets; 0.0977 mm^2^) of a parameter represents an animal in the graphic.

### 4.7. Sphingolipid Analysis

Sphingosine, S1P, and ceramides were analyzed by liquid chromatography coupled to triple quadrupole mass spectrometry (LC-MS/MS) using an ultra-performance liquid chromatography (UPLC) system (Nexera 40 series) and a triple quadrupole mass spectrometer LCMS-8050 (both from Shimadzu Deutschland, Duisburg, Germany). In total, 20 µL of a plasma sample was precipitated with the addition of 200 μL of methanol in vials. Approximately 20 mg of kidney tissue was homogenized with 2000 µL of methanol using the TissueLyser LT (Qiagen, Hilden, Germany) at 35 Hz for 3 min. After 3 days of incubation at −80 °C, the samples were centrifuged at 14,000× *g* for 10 min at 4 °C. The tissue supernatant was evaporated at 50 °C and 1000 rpm for 50 min in a rotational vacuum concentrator (Martin Christ Gefriertrocknungsanlagen, Osterode, Germany), and the vials refilled with 200 µL of methanol followed by shaking. Prior to processing, the methanol was spiked with internal standard (IS) solution, resulting in a final concentration of 297 nM 15:0 ceramide, 200 nM d17:1 S1P, and 40 nM d17:1 sphingosine in reconstituted samples. Plasma and renal tissue samples were analyzed with the S1P, sphingosine, or ceramide method. For the chromatographical separation of d18:1 and d17:1 S1P, the 2.1 mm I.D. × 150 mm L, 3 µm particle size Discovery HS F5-3 column (567503-U, Sigma Aldrich) was used, and for d18:1 and d17:1 sphingosine, a MultoHigh 100 RP 18-3µ 60 × 2 mm column (556201-1174, Chromatographie Service, Langerwehe, Germany) with intermittent runs for equilibration was used. Ceramide species were separated using the 2.1 × 150 mm 2.6 µm particle size C8 Kinetex LC Column (00F-4497-AN, Phenomenex, Aschaffenburg, Germany). Mass spectrometric detection was performed by multiple reactions monitoring (MRM). There is further information on the UPLC programs and solvents (Supplementary Table S4), LCMS-8050 settings (Supplementary Table S5), and recorded mass transitions of significantly changed analytes identified (Supplementary Table S6) in the Supplementary Materials. Mass spectrometry data were further processed with LabSolutions 5.91 and LabSolutions Insight 3.10 (Shimadzu Deutschland). The sphingolipid levels were quantified in a semi-quantitative manner relative to the according internal standard concentration. Values below the limit of detection were excluded from the analysis.

### 4.8. Statistical Analysis

Data were analyzed using GraphPad Prism 7.0 (Dotmatics, Boston, MA, USA). Data in the text and figures are expressed as median ± interquartile range of *n* observations. The Kruskal–Wallis test, followed by Dunn’s multiple comparisons test, was used to compare the Stx groups of each strain with their corresponding sham group, each mutant Stx group with the WT Stx group, and each mutant sham group with the WT sham group. Further possible group comparisons were not included in the statistical analysis because of missing biological relevance. A *p*-value < 0.05 was considered as significant.

## Data Availability

The raw data supporting the conclusions of this article will be made available by the authors on request.

## Supplementary Materials

The following supporting information can be downloaded at: https://www.mdpi.com/article/10.3390/ijms25147683/s1.

## References

[1] Gerber A., Karch H., Allerberger F., Verweyen H.M., Zimmerhackl L.B. (2002). Clinical course and the role of shiga toxin-producing *Escherichia coli* infection in the hemolytic-uremic syndrome in pediatric patients, 1997–2000, in Germany and Austria: A prospective study. J. Infect. Dis..

[2] Aldharman S.S., Almutairi S.M., Alharbi A.A., Alyousef M.A., Alzankrany K.H., Althagafi M.K., Alshalahi E.E., Al-Jabr K.H., Alghamdi A., Jamil S.F. (2023). The Prevalence and Incidence of Hemolytic Uremic Syndrome: A Systematic Review. Cureus.

[3] Adams N., Byrne L., Rose T., Adak B., Jenkins C., Charlett A., Violato M., O’Brien S., Whitehead M., Barr B. (2019). Sociodemographic and clinical risk factors for paediatric typical haemolytic uraemic syndrome: Retrospective cohort study. BMJ Paediatr. Open.

[4] Bruyand M., Mariani-Kurkdjian P., Le Hello S., King L.-A., Van Cauteren D., Lefevre S., Gouali M., Silva N.J.-D., Mailles A., Donguy M.-P. (2019). Paediatric haemolytic uraemic syndrome related to Shiga toxin-producing *Escherichia coli*, an overview of 10 years of surveillance in France, 2007 to 2016. Eurosurveillance.

[5] Myojin S., Michihata N., Shoji K., Takanashi J.-I., Matsui H., Fushimi K., Miyairi I., Yasunaga H. (2023). Prognostic factors among patients with Shiga toxin-producing *Escherichia coli* hemolytic uremic syndrome: A retrospective cohort study using a nationwide inpatient database in Japan. J. Infect. Chemother..

[6] Werber D., King L.A., Müller L., Follin P., Buchholz U., Bernard H., Rosner B., Ethelberg S., de Valk H., Höhle M. (2013). Associations of Age and Sex with the Clinical Outcome and Incubation Period of Shiga toxin-producing *Escherichia coli* O104:H4 Infections, 2011. Am. J. Epidemiol..

[7] Karmali M.A. (2004). Infection by Shiga Toxin-Producing *Escherichia coli*: An Overview. Mol. Biotechnol..

[8] Frank C., Werber D., Cramer J.P., Askar M., Faber M., an der Heiden M., Bernard H., Fruth A., Prager R., Spode A. (2011). Epidemic Profile of Shiga-Toxin–Producing *Escherichia coli* O104:H4 Outbreak in Germany. N. Engl. J. Med..

[9] Michino H., Araki K., Minami S., Takaya S., Sakai N., Miyazaki M., Ono A., Yanagawa H. (1999). Massive Outbreak of *Escherichia coli* O157: H7 Infection In Schoolchildren in Sakai City, Japan, Associated with Consumption of White Radish Sprouts. Am. J. Epidemiol..

[10] Rangel J.M., Sparling P.H., Crowe C., Griffin P.M., Swerdlow D.L. (2005). Epidemiology of *Escherichia coli* O157:H7 Outbreaks, United States, 1982–2002. Emerg. Infect. Dis..

[11] Adams N.L., Byrne L., Smith G.A., Elson R., Harris J.P., Salmon R., Smith R., O’brien S.J., Adak G.K., Jenkins C. (2016). Shiga Toxin-Producing *Escherichia coli* O157, England and Wales, 1983–2012. Emerg. Infect. Dis..

[12] Fakhouri F., Zuber J., Frémeaux-Bacchi V., Loirat C. (2017). Haemolytic uraemic syndrome. Lancet.

[13] Strockbine N.A., Marques L.R., Newland J.W., Smith H.W., Holmes R.K., O’Brien A.D. (1986). Two toxin-converting phages from *Escherichia coli* O157:H7 strain 933 encode antigenically distinct toxins with similar biologic activities. Infect. Immun..

[14] Keusch G.T. (1998). The Rediscovery of Shiga Toxin and Its Role in Clinical Disease. Jpn. J. Med. Sci. Biol..

[15] Karmali M.A., Steele B.T., Petric M., Lim C. (1983). Sporadic cases of haemolytic-uraemic syndrome associated with faecal cytotoxin and cytotoxin-producing *Escherichia coli* in stools. Lancet.

[16] Tesh V.L., Burris J.A., Owens J.W., Gordon V.M., Wadolkowski E.A., O’Brien A.D., Samuel J.E. (1993). Comparison of the relative toxicities of Shiga-like toxins type I and type II for mice. Infect. Immun..

[17] Scheutz F., Teel L.D., Beutin L., Piérard D., Buvens G., Karch H., Mellmann A., Caprioli A., Tozzoli R., Morabito S. (2012). Multicenter Evaluation of a Sequence-Based Protocol for Subtyping Shiga Toxins and Standardizing Stx Nomenclature. J. Clin. Microbiol..

[18] Fruth A., Lang C., Größl T., Garn T., Flieger A. (2024). Genomic surveillance of STEC/EHEC infections in Germany 2020 to 2022 permits insight into virulence gene profiles and novel O-antigen gene clusters. Int. J. Med. Microbiol..

[19] Seliga-Gąsior D., Sokól-Leszczyñska B., Krzysztoñ-Russjan J., Wierzbicka D., Stępieñ-Hołubczat K., Lewandowska P., Frankiewicz E., Cacko A., Leszczyñska B., Demkow U. (2024). Epidemiological Characteristics of Shiga Toxin-Producing *Escherichia coli* Responsible for Infections in the Polish Pediatric Population. Pol. J. Microbiol..

[20] Fernández-Brando R.J., Amaral M.M., Ciocchini A.E., Bentancor L.V., Trelles J.A., Da Rocha M., Landriel M., Ugarte M., Briones G., Ibarra C. (2017). Microbiological and serological control of *Escherichia coli* O157:H7 in kindergarten staff in Buenos Aires city and suburban areas. Medicina.

[21] Vonberg R.P., Höhle M., Aepfelbacher M., Bange F.C., Campos C.B., Claussen K., Christner M., Cramer J.P., Haller H., Hornef M. (2013). Duration of Fecal Shedding of Shiga Toxin–Producing *Escherichia coli* O104:H4 in Patients Infected During the 2011 Outbreak in Germany: A Multicenter Study. Clin. Infect. Dis..

[22] Hofer J., Giner T., Safouh H. (2014). Diagnosis and Treatment of the Hemolytic Uremic Syndrome Disease Spectrum in Developing Regions. Semin. Thromb. Hemost..

[23] Chan Y.S., Ng T.B. (2015). Shiga toxins: From structure and mechanism to applications. Appl. Microbiol. Biotechnol..

[24] Schüller S. (2011). Shiga Toxin Interaction with Human Intestinal Epithelium. Toxins.

[25] Jacewicz M., Clausen H., Nudelman E., Donohue-Rolfe A., Keusch G.T. (1986). Pathogenesis of shigella diarrhea. XI. Isolation of a shigella toxin-binding glycolipid from rabbit jejunum and HeLa cells and its identification as globotriaosylceramide. J. Exp. Med..

[26] Obrig T.G., Del Vecchio P.J., Brown J.E., Moran T.P., Rowland B.M., Judge T.K., Rothman S.W. (1988). Direct cytotoxic action of Shiga toxin on human vascular endothelial cells. Infect. Immun..

[27] Lingwood C.A. (1999). Verotoxin/Globotriaosyl Ceramide Recognition: Angiopathy, Angiogenesis and Antineoplasia. Biosci. Rep..

[28] Ling H., Boodhoo A., Hazes B., Cummings M.D., Armstrong G.D., Brunton J.L., Read R.J. (1998). Structure of the Shiga-like Toxin I B-Pentamer Complexed with an Analogue of Its Receptor Gb3. Biochemistry.

[29] Sandvig K., Garred Ø., Van Deurs B. (1997). Intracellular Transport and Processing of Protein Toxins Produced by Enteric Bacteria. Mech. Pathog. Enteric Dis..

[30] Ylinen E., Salmenlinna S., Halkilahti J., Jahnukainen T., Korhonen L., Virkkala T., Rimhanen-Finne R., Nuutinen M., Kataja J., Arikoski P. (2020). Hemolytic uremic syndrome caused by Shiga toxin–producing *Escherichia coli* in children: Incidence, risk factors, and clinical outcome. Pediatr. Nephrol..

[31] Zanchi C., Zoja C., Morigi M., Valsecchi F., Liu X.Y., Rottoli D., Locatelli M., Buelli S., Pezzotta A., Mapelli P. (2008). Fractalkine and CX3CR1 Mediate Leukocyte Capture by Endothelium in Response to Shiga Toxin. J. Immunol..

[32] Wang H., Rogers T.J., Paton J.C., Paton A.W. (2014). Differential Effects of *Escherichia coli* Subtilase Cytotoxin and Shiga Toxin 2 on Chemokine and Proinflammatory Cytokine Expression in Human Macrophage, Colonic Epithelial, and Brain Microvascular Endothelial Cell Lines. Infect. Immun..

[33] Brigotti M., Carnicelli D., Ravanelli E., Barbieri S., Ricci F., Bontadini A., Tozzi A.E., Scavia G., Caprioli A., Tazzari P.L. (2008). Interactions between Shiga toxins and human polymorphonuclear leukocytes. J. Leukoc. Biol..

[34] Guessous F., Marcinkiewicz M., Polanowska-Grabowska R., Kongkhum S., Heatherly D., Obrig T., Gear A.R.L. (2005). Shiga Toxin 2 and Lipopolysaccharide Induce Human Microvascular Endothelial Cells To Release Chemokines and Factors That Stimulate Platelet Function. Infect. Immun..

[35] Karpman D., Papadopoulou D., Nilsson K., Sjögren A.-C., Mikaelsson C., Lethagen S. (2001). Platelet activation by Shiga toxin and circulatory factors as a pathogenetic mechanism in the hemolytic uremic syndrome. Blood.

[36] Lentz E.K., Leyva-Illades D., Lee M.-S., Cherla R.P., Tesh V.L. (2011). Differential Response of the Human Renal Proximal Tubular Epithelial Cell Line HK-2 to Shiga Toxin Types 1 and 2. Infect. Immun..

[37] Márquez L.B., Velázquez N., Repetto H.A., Paton A.W., Paton J.C., Ibarra C., Silberstein C. (2014). Effects of *Escherichia coli* Subtilase Cytotoxin and Shiga Toxin 2 on Primary Cultures of Human Renal Tubular Epithelial Cells. PLoS ONE.

[38] Márquez L.B., Araoz A., Repetto H.A., Ibarra F.R., Silberstein C. (2016). Effects of shiga toxin 2 on cellular regeneration mechanisms in primary and three-dimensional cultures of human renal tubular epithelial cells. Microb. Pathog..

[39] Porubsky S., Federico G., Müthing J., Jennemann R., Gretz N., Büttner S., Obermüller N., Jung O., Hauser I.A., Gröne E. (2014). Direct acute tubular damage contributes to Shigatoxin-mediated kidney failure. J. Pathol..

[40] Viennet A., Pretalli J.-B., Vieux R., Nobili F. (2024). Kidney outcomes in Shiga toxin-associated hemolytic uremic syndrome in childhood: A retrospective single-center study from 1999 to 2017. Arch. Pediatr..

[41] Rosales A., Kuppelwieser S., Giner T., Hofer J., Khursigara M.R., Orth-Höller D., Borena W., Cortina G., Jungraithmayr T., Würzner R. (2024). Outcome 10 years after Shiga toxin-producing *E. coli* (STEC)-associated hemolytic uremic syndrome: Importance of long-term follow-up. Pediatr. Nephrol..

[42] Weigel C., Bellaci J., Spiegel S. (2023). Sphingosine-1-phosphate and its receptors in vascular endothelial and lymphatic barrier function. J. Biol. Chem..

[43] Romero D.J., Pescio L.G., Santacreu B.J., Mosca J.M., Sterin-Speziale N.B., Favale N.O. (2023). Sphingosine-1-phosphate receptor 2 plays a dual role depending on the stage of cell differentiation in renal epithelial cells. Life Sci..

[44] Greenspon J., Li R., Xiao L., Rao J.N., Sun R., Strauch E.D., Shea-Donohue T., Wang J.-Y., Turner D.J. (2010). Sphingosine-1-Phosphate Regulates the Expression of Adherens Junction Protein E-Cadherin and Enhances Intestinal Epithelial Cell Barrier Function. Dig. Dis. Sci..

[45] Sun G., Wang B., Wu X., Cheng J., Ye J., Wang C., Zhu H., Liu X. (2024). How do sphingosine-1-phosphate affect immune cells to resolve inflammation?. Front. Immunol..

[46] Van Brocklyn J.R., Williams J.B. (2012). The control of the balance between ceramide and sphingosine-1-phosphate by sphingosine kinase: Oxidative stress and the seesaw of cell survival and death. Comp. Biochem. Physiol. Part B Biochem. Mol. Biol..

[47] Pyne S., Pyne N.J. (2011). Translational aspects of sphingosine 1-phosphate biology. Trends Mol. Med..

[48] Pyne N.J., Adams D.R., Pyne S. (2017). Sphingosine Kinase 2 in Autoimmune/Inflammatory Disease and the Development of Sphingosine Kinase 2 Inhibitors. Trends Pharmacol. Sci..

[49] Spiegel S., Milstien S. (2011). The outs and the ins of sphingosine-1-phosphate in immunity. Nat. Rev. Immunol..

[50] Linke B., Schreiber Y., Zhang D.D., Pierre S., Coste O., Henke M., Suo J., Fuchs J., Angioni C., Ferreiros-Bouzas N. (2012). Analysis of sphingolipid and prostaglandin synthesis during zymosan-induced inflammation. Prostaglandins Other Lipid Mediat..

[51] Kharel Y., Huang T., Salamon A., Harris T.E., Santos W.L., Lynch K.R. (2020). Mechanism of sphingosine 1-phosphate clearance from blood. Biochem. J..

[52] Wilkerson J.L., Stiles M.A., Gurley J.M., Grambergs R.C., Gu X., Elliott M.H., Proia R.L., Mandal N.A. (2019). Sphingosine Kinase-1 Is Essential for Maintaining External/Outer Limiting Membrane and Associated Adherens Junctions in the Aging Retina. Mol. Neurobiol..

[53] Thuy A.V., Reimann C.-M., Ziegler A.C., Gräler M.H. (2022). The Impact of Sphingosine Kinases on Inflammation-Induced Cytokine Release and Vascular Endothelial Barrier Integrity. Int. J. Mol. Sci..

[54] Schwalm S., Beyer S., Frey H., Haceni R., Grammatikos G., Thomas D., Geisslinger G., Schaefer L., Huwiler A., Pfeilschifter J. (2017). Sphingosine Kinase-2 Deficiency Ameliorates Kidney Fibrosis by Up-Regulating Smad7 in a Mouse Model of Unilateral Ureteral Obstruction. Am. J. Pathol..

[55] Coldewey S.M., Benetti E., Collino M., Pfeilschifter J., Sponholz C., Bauer M., Huwiler A., Thiemermann C. (2016). Elevation of serum sphingosine-1-phosphate attenuates impaired cardiac function in experimental sepsis. Sci. Rep..

[56] Weigel C., Hüttner S.S., Ludwig K., Krieg N., Hofmann S., Schröder N.H., Robbe L., Kluge S., Nierhaus A., Winkler M.S. (2020). S1P lyase inhibition protects against sepsis by promoting disease tolerance via the S1P/S1PR3 axis. EBioMedicine.

[57] Xie D., Hu G., Chen C., Ahmadinejad F., Wang W., Li P.-L., Gewirtz D.A., Li N. (2022). Loss of sphingosine kinase 2 protects against cisplatin-induced kidney injury. Am. J. Physiol. Ren. Physiol..

[58] Ghosh M., Thangada S., Dasgupta O., Khanna K.M., Yamase H.T., Kashgarian M., Hla T., Shapiro L.H., Ferrer F.A. (2018). Cell-intrinsic sphingosine kinase 2 promotes macrophage polarization and renal inflammation in response to unilateral ureteral obstruction. PLoS ONE.

[59] Jo S.-K., Bajwa A., Ye H., Vergis A.L., Awad A.S., Kharel Y., Lynch K.R., Okusa M.D. (2009). Divergent roles of sphingosine kinases in kidney ischemia–reperfusion injury. Kidney Int..

[60] Zhang X., Wang W., Ji X.-Y., Ritter J.K., Li N. (2019). Knockout of Sphingosine Kinase 1 Attenuates Renal Fibrosis in Unilateral Ureteral Obstruction Model. Am. J. Nephrol..

[61] Dennhardt S., Pirschel W., Wissuwa B., Daniel C., Gunzer F., Lindig S., Medyukhina A., Kiehntopf M., Rudolph W.W., Zipfel P.F. (2018). Modeling Hemolytic-Uremic Syndrome: In-Depth Characterization of Distinct Murine Models Reflecting Different Features of Human Disease. Front. Immunol..

[62] Russo L.M., Melton-Celsa A.R., Smith M.A., O’Brien A.D. (2014). Oral Intoxication of Mice with Shiga Toxin Type 2a (Stx2a) and Protection by Anti-Stx2a Monoclonal Antibody 11E10. Infect. Immun..

[63] Kröller S., Wissuwa B., Dennhardt S., Krieg N., Thiemermann C., Daniel C., Amann K., Gunzer F., Coldewey S.M. (2023). Bruton’s tyrosine kinase inhibition attenuates disease progression by reducing renal immune cell invasion in mice with hemolytic-uremic syndrome. Front. Immunol..

[64] Pirschel W., Mestekemper A.N., Wissuwa B., Krieg N., Kröller S., Daniel C., Gunzer F., Tolosano E., Bauer M., Amann K. (2022). Divergent roles of haptoglobin and hemopexin deficiency for disease progression of Shiga-toxin–induced hemolytic-uremic syndrome in mice. Kidney Int..

[65] Kaplan B.S. (1998). Shiga toxin-induced tubular injury in hemolytic uremic syndrome. Kidney Int..

[66] Rutjes N.W., Binnington B.A., Smith C.R., Maloney M.D., Lingwood C.A. (2002). Differential tissue targeting and pathogenesis of verotoxins 1 and 2 in the mouse animal model. Kidney Int..

[67] Shimizu M. (2019). Pathogenic functions and diagnostic utility of cytokines/chemokines in EHEC-HUS. Pediatr. Int..

[68] Winkler M.S., Märtz K.B., Nierhaus A., Daum G., Schwedhelm E., Kluge S., Gräler M.H. (2019). Loss of sphingosine 1-phosphate (S1P) in septic shock is predominantly caused by decreased levels of high-density lipoproteins (HDL). J. Intensive Care.

[69] Xiong Y., Yang P., Proia R.L., Hla T. (2014). Erythrocyte-derived sphingosine 1-phosphate is essential for vascular development. J. Clin. Investig..

[70] Vu T.M., Ishizu A.-N., Foo J.C., Toh X.R., Zhang F., Whee D.M., Torta F., Cazenave-Gassiot A., Matsumura T., Kim S. (2017). Mfsd2b is essential for the sphingosine-1-phosphate export in erythrocytes and platelets. Nature.

[71] Sun K., Zhang Y., D’alessandro A., Nemkov T., Song A., Wu H., Liu H., Adebiyi M., Huang A., Wen Y.E. (2016). Sphingosine-1-phosphate promotes erythrocyte glycolysis and oxygen release for adaptation to high-altitude hypoxia. Nat. Commun..

[72] Ullah M., Basile D.P. (2019). Role of Renal Hypoxia in the Progression from Acute Kidney Injury to Chronic Kidney Disease. Semin. Nephrol..

[73] Peng X., Hassoun P.M., Sammani S., McVerry B.J., Burne M.J., Rabb H., Pearse D., Tuder R.M., Garcia J.G.N. (2004). Protective Effects of Sphingosine 1-Phosphate in Murine Endotoxin-induced Inflammatory Lung Injury. Am. J. Respir. Crit. Care Med..

[74] McVerry B.J., Peng X., Hassoun P.M., Sammani S., Simon B.A., Garcia J.G.N. (2004). Sphingosine 1-Phosphate Reduces Vascular Leak in Murine and Canine Models of Acute Lung Injury. Am. J. Respir. Crit. Care Med..

[75] Kharel Y., Raje M., Gao M., Gellett A.M., Tomsig J.L., Lynch K.R., Santos W.L. (2012). Sphingosine kinase type 2 inhibition elevates circulating sphingosine 1-phosphate. Biochem. J..

[76] Di A., Kawamura T., Gao X.-P., Tang H., Berdyshev E., Vogel S.M., Zhao Y.-Y., Sharma T., Bachmaier K., Xu J. (2010). A Novel Function of Sphingosine Kinase 1 Suppression of JNK Activity in Preventing Inflammation and Injury. J. Biol. Chem..

[77] Tauseef M., Kini V., Knezevic N., Brannan M., Ramchandaran R., Fyrst H., Saba J., Vogel S.M., Malik A.B., Mehta D. (2008). Activation of Sphingosine Kinase-1 Reverses the Increase in Lung Vascular Permeability Through Sphingosine-1-Phosphate Receptor Signaling in Endothelial Cells. Circ. Res..

[78] Zhang Z.-S., Liu Y.-Y., He S.-S., Bao D.-Q., Wang H.-C., Zhang J., Peng X.-Y., Zang J.-T., Zhu Y., Wu Y. (2023). Pericytes protect rats and mice from sepsis-induced injuries by maintaining vascular reactivity and barrier function: Implication of miRNAs and microvesicles. Mil. Med. Res..

[79] Patyna S., Büttner S., Eckes T., Obermüller N., Bartel C., Braner A., Trautmann S., Thomas D., Geiger H., Pfeilschifter J. (2019). Blood ceramides as novel markers for renal impairment in systemic lupus erythematosus. Prostaglandins Other Lipid Mediat..

[80] Dupre T.V., Siskind L.J. (2018). The role of sphingolipids in acute kidney injury. Adv. Biol. Regul..

[81] Basnakian A.G., Ueda N., Hong X., Galitovsky V.E., Yin X., Shah S.V. (2005). Ceramide synthase is essential for endonuclease-mediated death of renal tubular epithelial cells induced by hypoxia-reoxygenation. Am. J. Physiol. Ren. Physiol..

[82] Ueda N., Camargo S.M.R., Hong X., Basnakian A.G., Walker P.D., Shah S.V. (2001). Role of Ceramide Synthase in Oxidant Injury to Renal Tubular Epithelial Cells. J. Am. Soc. Nephrol..

[83] Zager R.A., Conrad D.S., Burkhart K. (1998). Ceramide accumulation during oxidant renal tubular injury. J. Am. Soc. Nephrol..

[84] Drexler Y., Molina J., Mitrofanova A., Fornoni A., Merscher S. (2020). Sphingosine-1-Phosphate Metabolism and Signaling in Kidney Diseases. J. Am. Soc. Nephrol..

[85] Eresch J., Stumpf M., Koch A., Vutukuri R., Ferreirós N., Schreiber Y., Schröder K., Devraj K., Popp R., Huwiler A. (2018). Sphingosine Kinase 2 Modulates Retinal Neovascularization in the Mouse Model of Oxygen-Induced Retinopathy. Investig. Ophthalmol. Vis. Sci..

[86] Argyle J.C., Hogg R.J., Pysher T.J., Silva F.G., Siegler R.L. (1990). A clinicopathological study of 24 children with hemolytic uremic syndrome. Pediatr. Nephrol..

[87] Inward C.D., Howie A.J., Fitzpatrick M.M., Rafaat F., Milford D.V., Taylor C.M. (1997). Renal histopathology in fatal cases of diarrhoea-associated haemolytic uraemic syndrome. Pediatr. Nephrol..

[88] Hughes A.K., Stricklett P.K., Kohan D.E. (1998). Shiga toxin-1 regulation of cytokine production by human proximal tubule cells. Kidney Int..

[89] Wang C., Li Q., Lv J., Sun X., Cao Y., Yu K., Miao C., Zhang Z.-S., Yao Z., Wang Q. (2019). Alpha-hemolysin of uropathogenic *Escherichia coli* induces GM-CSF-mediated acute kidney injury. Mucosal Immunol..

[90] Cyster J.G., Schwab S.R. (2012). Sphingosine-1-Phosphate and Lymphocyte Egress from Lymphoid Organs. Annu. Rev. Immunol..

[91] Bajwa A., Huang L., Kurmaeva E., Ye H., Dondeti K.R., Chroscicki P., Foley L.S., Balogun Z.A., Alexander K.J., Park H. (2016). Sphingosine Kinase 2 Deficiency Attenuates Kidney Fibrosis via IFN-γ. J. Am. Soc. Nephrol..

[92] Samy E.T., Meyer C.A., Caplazi P., Langrish C.L., Lora J.M., Bluethmann H., Peng S.L. (2007). Cutting Edge: Modulation of Intestinal Autoimmunity and IL-2 Signaling by Sphingosine Kinase 2 Independent of Sphingosine 1-Phosphate. J. Immunol..

[93] Keepers T.R., Gross L.K., Obrig T.G. (2007). Monocyte Chemoattractant Protein 1, Macrophage Inflammatory Protein 1α, and RANTES Recruit Macrophages to the Kidney in a Mouse Model of Hemolytic-Uremic Syndrome. Infect. Immun..

[94] Griffin P.M., Ostroff S.M., Tauxe R.V., Greene K.D., Wells J.G., Lewis J.H., Blake P.A. (1988). Illnesses Associated with *Escherichia coli* 0157:H7 Infections. Ann. Intern. Med..

[95] Shimizu T., Ohta Y., Noda M. (2009). Shiga Toxin 2 Is Specifically Released from Bacterial Cells by Two Different Mechanisms. Infect. Immun..

[96] Allende M.L., Sasaki T., Kawai H., Olivera A., Mi Y., van Echten-Deckert G., Hajdu R., Rosenbach M., Keohane C.A., Mandala S. (2004). Mice Deficient in Sphingosine Kinase 1 Are Rendered Lymphopenic by FTY720. J. Biol. Chem..

[97] Mizugishi K., Yamashita T., Olivera A., Miller G.F., Spiegel S., Proia R.L. (2005). Essential Role for Sphingosine Kinases in Neural and Vascular Development. Mol. Cell. Biol..

[98] Faul F., Erdfelder E., Lang A.-G., Buchner A. (2007). G*Power 3: A flexible statistical power analysis program for the social, behavioral, and biomedical sciences. Behav. Res. Methods.

