# Supplementary material for: Deletion of Sphingosine Kinase 2 Attenuates Acute Kidney Injury in Mice with Hemolytic-Uremic Syndrome
Source: Int J Mol Sci. 2024 Jul 12;25(14):7683. doi: 10.3390/ijms25147683 (PMC11277509; doi:10.3390/ijms25147683)
Supplement: Supplementary file 1 [file ijms-25-07683-s001.zip › 2024-07-12_Supplementary_Material_Tables.pdf]

Deletion of Sphingosine Kinase 2 Attenuates Acute Kidney Injury in Mice with Hemolytic–Uremic Syndrome

Tina Müller [1,2,†], Nadine Krieg [1,2,†], Antonia I. Lange–Polovinkin [1,2], Bianka Wissuwa [1,2], Markus H. Gräler [1,3,4], Sophie Dennhardt [1,2] and Sina M. Coldewey [1,2,4,*]

[1] Department of Anesthesiology and Intensive Care Medicine, Jena University Hospital, 07743 Jena, Germany; tina.mueller2@med.uni–jena.de (T.M.); nadine.krieg@med.uni–jena.de (N.K.)
[2] ZIK Septomics Research Center, Jena University Hospital, 07743 Jena, Germany
[3] Center for Molecular Biomedicine (CMB) and Center for Sepsis Control and Care (CSCC), Jena University Hospital, 07743 Jena, Germany
[4] Center for Sepsis Control and Care (CSCC), Jena University Hospital, 07743 Jena, Germany
* Correspondence: sina.coldewey@med.uni–jena.de
† These authors have contributed equally to this work.

Supplementary Material

Table of Contents

**Supplementary Tables**	
**Supplementary Table S1**	Hemolytic–uremic syndrome (HUS) score
**Supplementary Table S2**	Commercial kits
**Supplementary Table S3**	Primary and secondary antibodies used for immunohistochemistry
**Supplementary Table S4**	UPLC programs
**Supplementary Table S5**	Mass spectrometer (LCMS 8050) settings
**Supplementary Table S6**	Mass transitions for sphingosine–1–phosphate, sphingosine and ceramides
**Supplementary Table S7**	Cytokine analysis in plasma of WT, SphK1$^{-/-}$ and SphK2$^{-/-}$ mice with experimental HUS on day 5

Supplementary Tables

Supplementary Table S1. Hemolytic–uremic syndrome (HUS) score. Each criterion was monitored three times daily. Grade of disease was calculated from the sum of all points of all criteria. Additionally, after two times consecutive score 3, the mice were checked one more time. Grade 1 = no signs of illness (6 points); grade 2 = low–grade (7–10 points); grade 3 = mid–grade (11–18 points); grade 4 = high–grade (≥ 19 points); grade 5 = dead.

Points per criterion	I. activity	II. reaction	III. posture	IV. general symptoms	V. neurological symptoms	VI. fur
1	active, strong	curious, fast movements	normal	none	none	shiny, even
2 for I, II, VI; 1 for III, IV, V	lower activity, occasional interruptions	reduced attention, appropriate reaction	normal	none	none	blunt, adjacent
3	markedly reduced	reduced attention, delayed reaction	slightly hunche d	loss of 10–20% BW in 48 h	beginning hind limb clasping	blunt, slightly ruffled
4	lethargic, no movements	none	strong hunch	loss of > 20% BW in 48 h	pronounced hind limb clasping	blunt, strong piloerection

Termination criteria: reaching an overall HUS score of 4 at two consecutive observation points, paraparesis that is likely to cause difficulties with food and water intake, dehydration, dark discoloration of the lower abdomen or scrotum, seizures/apathy, weight loss >20% within 48 hours, refusal of food and water intake, distinct reduced respiratory rate.

Supplementary Table S2. Commercial kits.

Target/method	Product name	Company	Cat. No.	Sample dilution
In vitro Toxicology Assay	IN VITRO TOXICOLOGY ASSAY KIT NEUTRAL RED BASED	Merck KGaA	TOX4	
Neutrophil gelatinase−associated lipocalin (NGAL)	Mouse NGAL (Lipocalin−2) ELISA Kit BioLegend, Inc.	BioLegend, Inc.	443707	sham: 1:200<br>Stx: 1:200/1:1000
Urea	Urea Assay Kit	Abcam plc.	Ab83362	1:200, except SphK1$^{-/-}$ Stx 1:400
PAS	PAS Staining Kit	Carl Roth GmbH	HP01.1	
Blocking	Avidin/Biotin Blocking Kit	Vector Laboratories, Inc.	SP−2001	
Horseradish Peroxidase conjugation	VectaStain Elite ABC Kit (Standard)	Vector Laboratories, Inc.	PK−6100	
	ImmPACT DAB Peroxidase HRP Substrate	Vector Laboratories, Inc.	SK−4105	

Supplementary Table S3. Primary and secondary antibodies used for immunohistochemistry.

Antibody	Company	Blocking	Dilution	Cat. No.
Polyclonal goat anti−KIM−1	R&D Systems, Inc.	1% milk powder in TRIS buffer, 1h, RT	1:1000 in TRIS buffer + 1% BSA*	AF1817
Monoclonal rat anti−CD31	Dianova GmbH	−	1:100 in TRIS buffer + 1% BSA	DIA−310
Monoclonal rat anti−F4−80	Bio−Rad Laboratories, Inc	1:5 rabbit serum:1% milk powder in TRIS buffer, 30 min, RT	1:100 in TRIS buffer + 1% BSA	MCA497
Monoclonal rabbit anti−Ki−67	Thermo Fisher Scientific, Inc.	1:5 rabbit serum:1% milk powder in TRIS buffer, 30 min, RT	1:200 in TRIS buffer + 1% BSA	12683697
Biotinylated anti−rabbit IgG (H+L)	Vector Laboratories, Inc.		1:200 in TRIS buffer + 1% BSA	BA−1000
Biotinylated anti−goat IgG (H+L)	Vector Laboratories, Inc.		1:500 in TRIS buffer + 1% BSA	BA−5000
Biotinylated anti−rat IgG (H+L)	Vector Laboratories, Inc.		1:200 in TRIS buffer + 1% BSA + 2% mouse serum	BA−4001

Abbreviations: BSA, bovine serum albumin; Tris, tris(hydroxymethyl)aminomethan; RT, room temperature.

Supplementary Table S4. UPLC programs. Solvent A: 0.1% formic acid in water, solvent B: 0.1% formic acid in acetonitrile, solvent C: methanol. The column oven temperature was set to 50 °C.

**Time [min]**	**Flow [mL/min]**	**Solvent A concentration [%]**	**Solvent B concentration [%]**	**Solvent C concentration [%]**
Sphingosine–1–phosphate				
0.0	0.25	100	0	
2.0	0.25	100	0	
5.0	0.25	75	25	
11.0	0.25	65	35	
15.0	0.25	5	95	
20.0	0.25	5	90	
20.1	0.25	90	10	
25.0	Stop			
Sphingosine				
0.00	0.4	90		10
0.01	0.4	0		100
3.00	0.4	0		100
5.00	0.8	0		100
7.00	0.8	0		100
7.01	0.8	90		10
7.80	0.8	90		10
8.30	0.3	90		10
9.50	0.3	90		10
9.51	Stop			
Ceramides				
0.00	0.2	90	10	
10.0	0.2	75	25	
20.0	0.2	65	35	
40.0	0.2	25	75	
40.2	0.2	5	95	
70.0	0.2	5	95	
70.2	0.2	90	10	
82.0	Stop			

Supplementary Table S5. Mass spectrometer (LCMS 8050) settings.

**Source conditions**	**Parameters**
Nebulizing gas flow rate	3.0 L/min
Heating gas flow rate	10.0 L/min
Drying gas flow rate	10.0 L/min
Collision–induced dissociation gas pressure	180 kPa
Interface temperature	300°C
Desolvation line temperature	250 °C
Heat block temperature	400 °C
Ionization mode	Electrospray ionization (ESI)
Nebulizing gas flow rate	5.0 L/min
Drying gas flow rate	10.0 L/min
Collision–induced dissociation gas pressure	230 kPa
Interface temperature	350 °C
Desolvation line temperature	250 °C
Heat block temperature	200 °C
Ionization mode	Atmosphere pressure chemical ionization (APCI)

Supplementary Table S6. Mass transitions for sphingosine–1–phosphate, sphingosine and ceramides. The target ion shows the multiple reaction monitoring (MRM) transitions, the ionization polarity (IP) shows the ionization mode of the ESI/APCI source and the internal standard (IS) column assigns the number of the internal standard to the compounds with which they were evaluated.

**Compound**	**Target Ion**	**IP**	**Sample injection volume [µL]**	**IS**
d18:1 sphingosine–1–phosphate	380.3>264.4	ESI(+)	20	1
d17:1 sphingosine–1–phosphate (IS)	366.3>250.4	ESI(+)	20	1
d18:1 sphingosine	300.4>282.4	ESI(+)	5	2
d17:1 sphingosine (IS)	286.3>268.4	ESI(+)	5	2
d18:1/16:0 ceramide	538.7>264.4	APCI(+)	10	3
d18:1/18:0 ceramide	566.7>264.4	APCI(+)	10	3
d18:1/20:0 ceramide	594.7>264.4	APCI(+)	10	3
d18:1/22:0 ceramide	622.8>264.4	APCI(+)	10	3
d18:1/24:0 ceramide	650.9>264.4	APCI(+)	10	3
d18:1/24:1 ceramide	648.9>264.4	APCI(+)	10	3
15:0 ceramide (IS)	524.5>264.4	APCI(+)	10	3

Abbreviations: ESI, electrospray ionization; APCI, atmosphere pressure chemical ionization

Supplementary Table S7. Cytokine analysis in plasma of WT, SphK1$^{-/-}$ and SphK2$^{-/-}$ mice with experimental HUS on day 5. Summarized are medians, 1. and 3. quartile of plasma cytokine levels (pg/ml) of statistically not significant group comparisons. WT sham: $n = 4$, WT Stx: $n = 5$, SphK1$^{-/-}$ sham: $n = 5$, SphK1$^{-/-}$ Stx: $n = 6$, SphK2$^{-/-}$ sham: $n = 5$, SphK2$^{-/-}$ Stx: $n = 6$. Kruskal–Wallis test + Dunn's multiple comparison test: *P < 0.05. Q1: 25% percentile, Q3: 75% percentile.

**Cytokine**	**WT sham** median (Q1–Q3)	**WT Stx** median (Q1–Q3)	**SphK1$^{-/-}$ sham** median (Q1–Q3)	**SphK1$^{-/-}$ Stx** median (Q1–Q3)	**SphK2$^{-/-}$ sham** median (Q1–Q3)	**SphK2$^{-/-}$ Stx** median (Q1–Q3)
BCA−1	559.8 (486.1–718.2)	955.2 (621.1–1249)	773.8 (534.2–1153)	1218 (747.2–1916)	613.4 (541.7–881.9)	1185 (911.4–1615)
Eotaxin	876.0 (462.7–1405)	1236 (928.3–1452)	815.8 (653.8–957.7)	1111 (803.4–1518)	690.2 (646.1–827.6)	892.0 (811–950.4)
Fractalkine	129.0 (96.01–135.4)	167.7 (152.8–191.9)	148.0 (134.4–164.6)	185.5 (172.6–203.1)	164.9 (138.8–176.1)	191.8 (162.4–221.1)
TARC	0 (0–7.3)	4.8 (0–7.5)	7.3 (0–13.1)	11.9 (8.4–15.7)	0 (0–12.2)	11.8 (6.84–16.3)
CTACK	610.4 (408.1–799.6)	3871 (390.7–7123)	2774 (229.2–13407)	4456 (0–8277)	1512 (636.1–2072)	1048 (0–3725)
ENA−78	88.9 (42.1–168.2)	153.0 (107.1–168.8)	153.0 (147.4–334)	123.6 (106.4–201)	204.2 (119.3–551.3)	163.9 (135.8–655.2)
Eotaxin−2	1573 (913.1–2039)	2098 (1450–3062)	2437 (2197–2821)	2198 (1794–2803)	2492 (2169–2785)	2462 (2039–2708)
I−309	10.9 (9.9–11.2)	28.2 (5.3–36.1)	9.0 3.9–30.4	40.20 (19.2–178.9)	28.84 (8.6–32.7)	22.34 (15.1–29.3)
IFNγ	4.5 (3–8.2)	9.3 (4.3–17.4)	16.3 (13.4–40.2)	20.7 (12.1–40.7)	11.9 (3.6–27.7)	13.2 (9.5–18.2)
IL−1ß	0.0 (0–0)	0.0 (0–109.9)	0.0 (0–0)	16.9 (0–62.4)	0.0 (0–100.2)	16.9 (0–140.4)
IL−2	0.0 (0–0)	0.0 (0–0)	0.0 (0–0)	0.0 (0–0)	0.0 (0–0.4)	0.0 (0–1.3)
IL−4	8.4 (8.4–15.7)	11.1 (8.2–23.2)	0.0 (0–10.8)	18.8 (9–29.3)	19.9 (19.9–32)	28.7 (18.4–35.3)
IL−10	0.0 (0–0)	0.0 (0–30.4)	0.0 (0–15.7)	85.6 (23.5–94)	31.3 (5.4–213.6)	59.2 (16–139.6)
IL−16	830.9 (646.7–1155)	1806 (663.6–2212)	595.9 (169–1685)	807.2 (0–1820)	953.5 (576.5–1247)	929.2 (495.6–1171)
I−Tac	0.0 (0–0)	0.0 (0–0)	0.0 (0–0)	0.0 (0–0)	0.0 (0–1378)	0.0 (0–1278)
MCP−1	0.0 (0–0)	0.0 (0–0)	0.0 (0–0)	0.0 (0–0)	0.0 (0–32.1)	0.0 (0–47.3)
MCP−3	16.9 (14.1–20.4)	25.5 (20.1–30.9)	25.1 (20.7–29.2)	33.7 (32.1–36)	21.8 (19.6–25)	26.1 (21.9–30.3)
MIP−1ß	0.0 (0–0)	0.0 (0–0)	0.0 (0–0)	0.0 (0–0)	0.0 (0–0)	0.0 (0–0)
MIP−3alpha	31.0 (11.9–35.4)	29.8 (10–89.9)	57.3 (5.4–160.7)	23.6 (0–152.5)	16.1 (10.1–19.3)	19.5 (3.4–27)
SCYB16	228.1 (165.5–346.3)	178.8 (81.6–277.1)	162.0 (50.9–319.5)	72.2 (0–155.9)	301.5 (151.6–326.7)	292.2 (142–398.8)
SDF−1alpha	183.4 (125.3–302.8)	147.4 (128.6–222.1)	222.1 (153.3–318.5)	219.7 (212.3–230.3)	212.3 (197.1–465)	318.4 (211.9–435.7)
TNF−alpha	34.1 (24.1–45.3)	27.4 (21.6–72.8)	40.8 (23.1–59.7)	62 (43.8–86.6)	39.3 (14.4–78.1)	40.8 (31.8–71.2)

Abbreviations: BCA, B cell–attracting chemokine; CTACK, cutaneous T cell–attracting chemokine; ENA, epithelial–derived neutrophil–activating protein; GM–CSF, granulocyte–macrophage colony–stimulating factor; HUS, hemolytic–uremic

syndrome; I–Tac, interferon–inducible T cell alpha chemoattractant; IFN, interferon; IL, interleukin; IP, interferon–gamma induced protein; KC, keratinocyte–derived chemokine; MCP, monocyte chemoattractant protein; MDC, macrophage–derived chemokine; MIP, macrophage inflammatory protein; SCYB, small–inducible cytokine, SD, standard deviation; SDF, stromal cell–derived factor; SphK, sphingosine kinase; Stx, shiga toxin; TARC, thymus– and activation–regulated chemokine; TNF, tumor necrosis factor; WT, wild type.
