# Supplementary figures and images for: Deletion of Sphingosine Kinase 2 Attenuates Acute Kidney Injury in Mice with Hemolytic-Uremic Syndrome
Source: Int J Mol Sci. 2024 Jul 12;25(14):7683. doi: 10.3390/ijms25147683 (PMC11277509; doi:10.3390/ijms25147683)

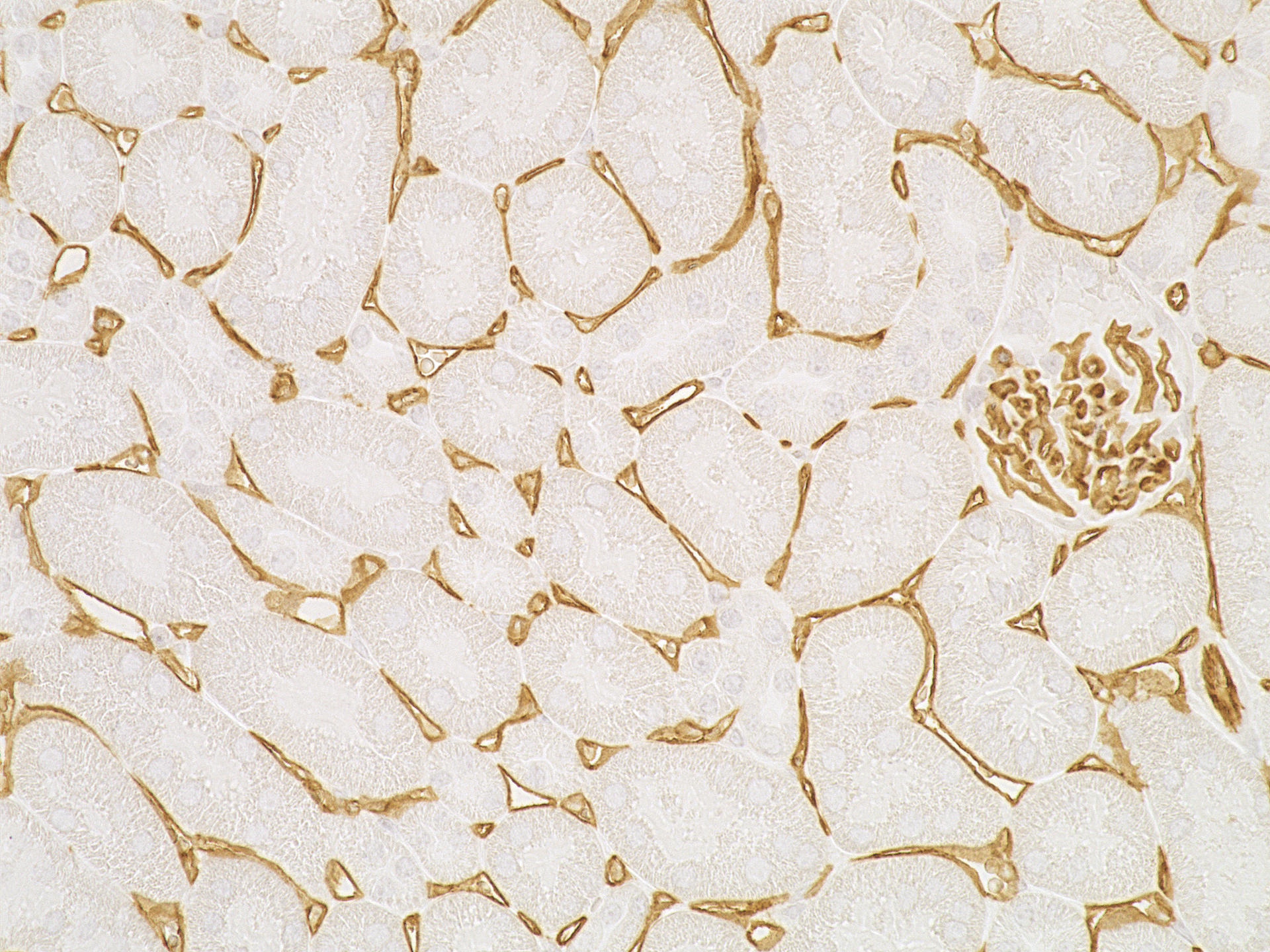

Supplement: Supplementary file 1 [file ijms-25-07683-s001.zip › Supplementary_Material_Microscopy_Images/SupplMat_CD31/SphK1_sham_028_CD31_2.gif]

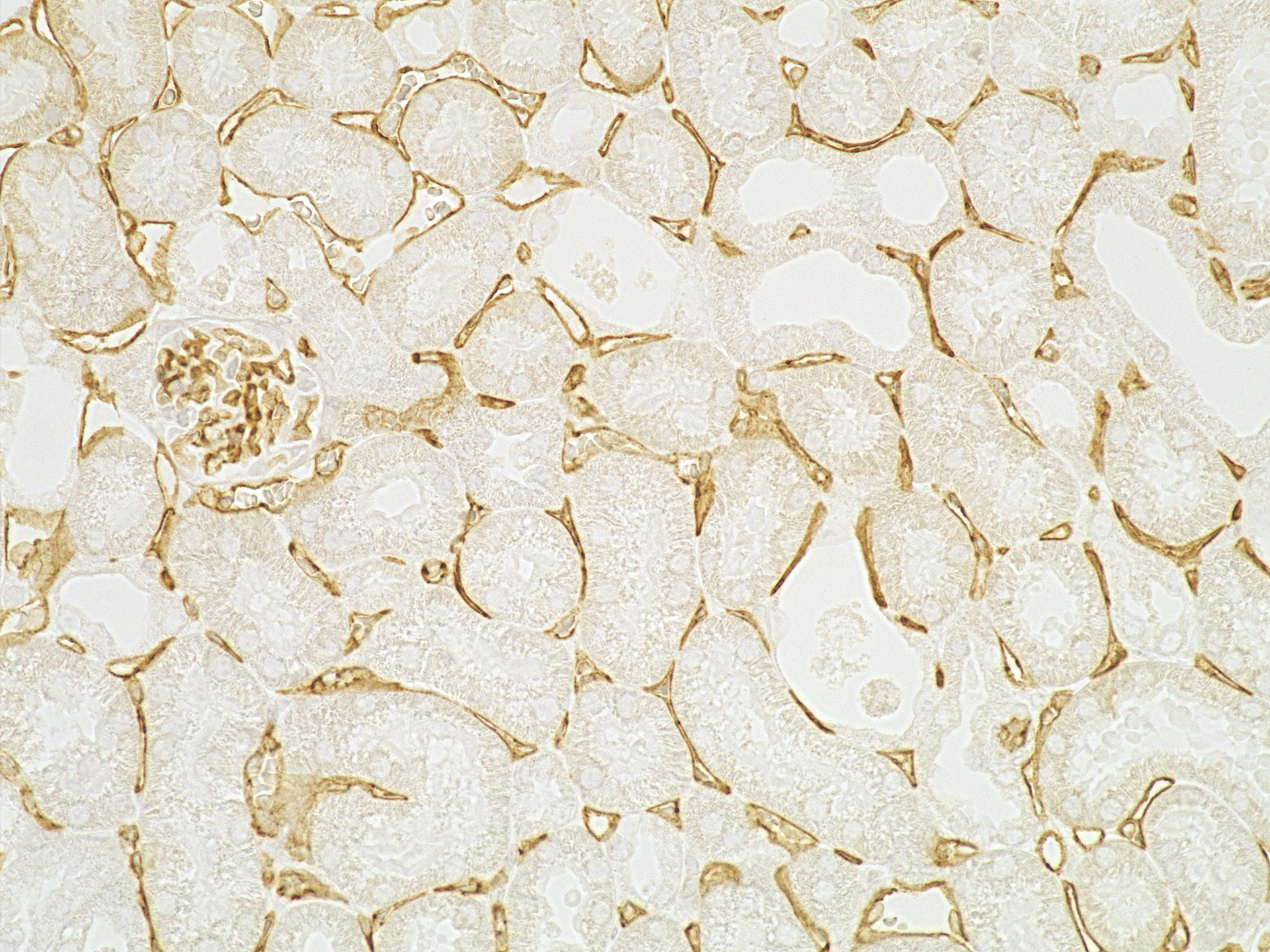

Supplement: Supplementary file 1 [file ijms-25-07683-s001.zip › Supplementary_Material_Microscopy_Images/SupplMat_CD31/SphK1_Stx_018_CD31_2.gif]

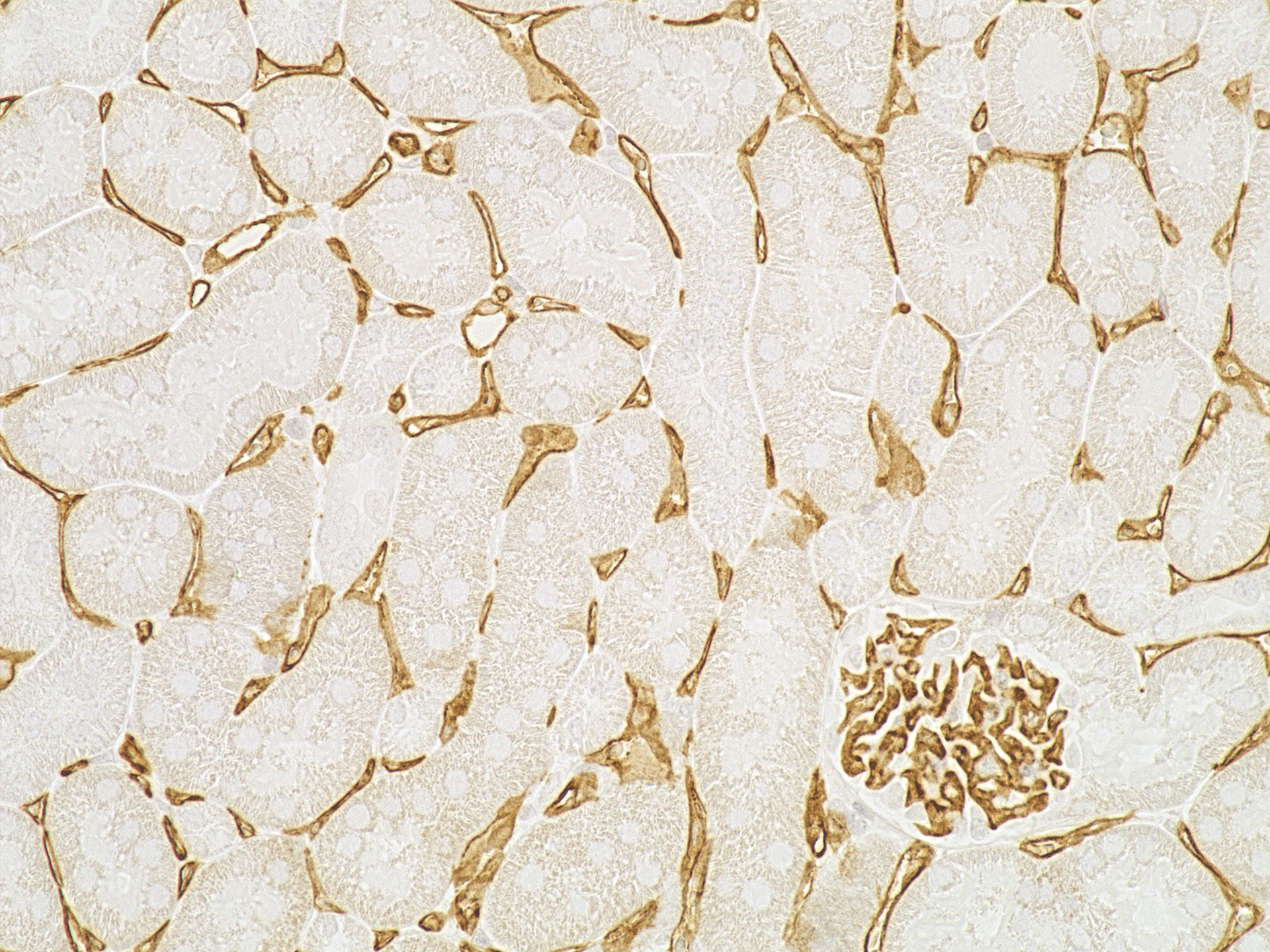

Supplement: Supplementary file 1 [file ijms-25-07683-s001.zip › Supplementary_Material_Microscopy_Images/SupplMat_CD31/SphK2_sham_019_CD31_8.gif]

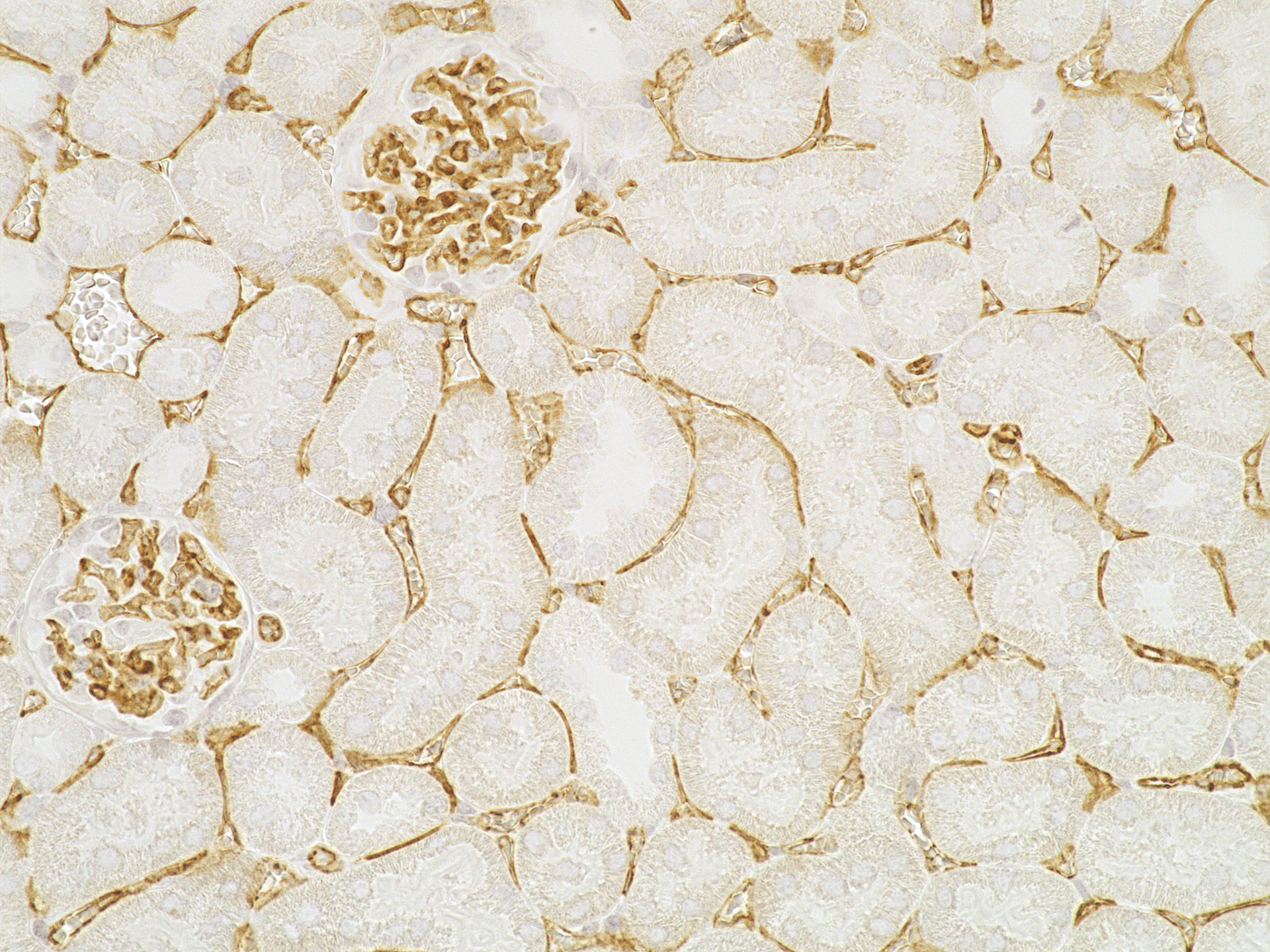

Supplement: Supplementary file 1 [file ijms-25-07683-s001.zip › Supplementary_Material_Microscopy_Images/SupplMat_CD31/SphK2_Stx_026_CD31_5.gif]

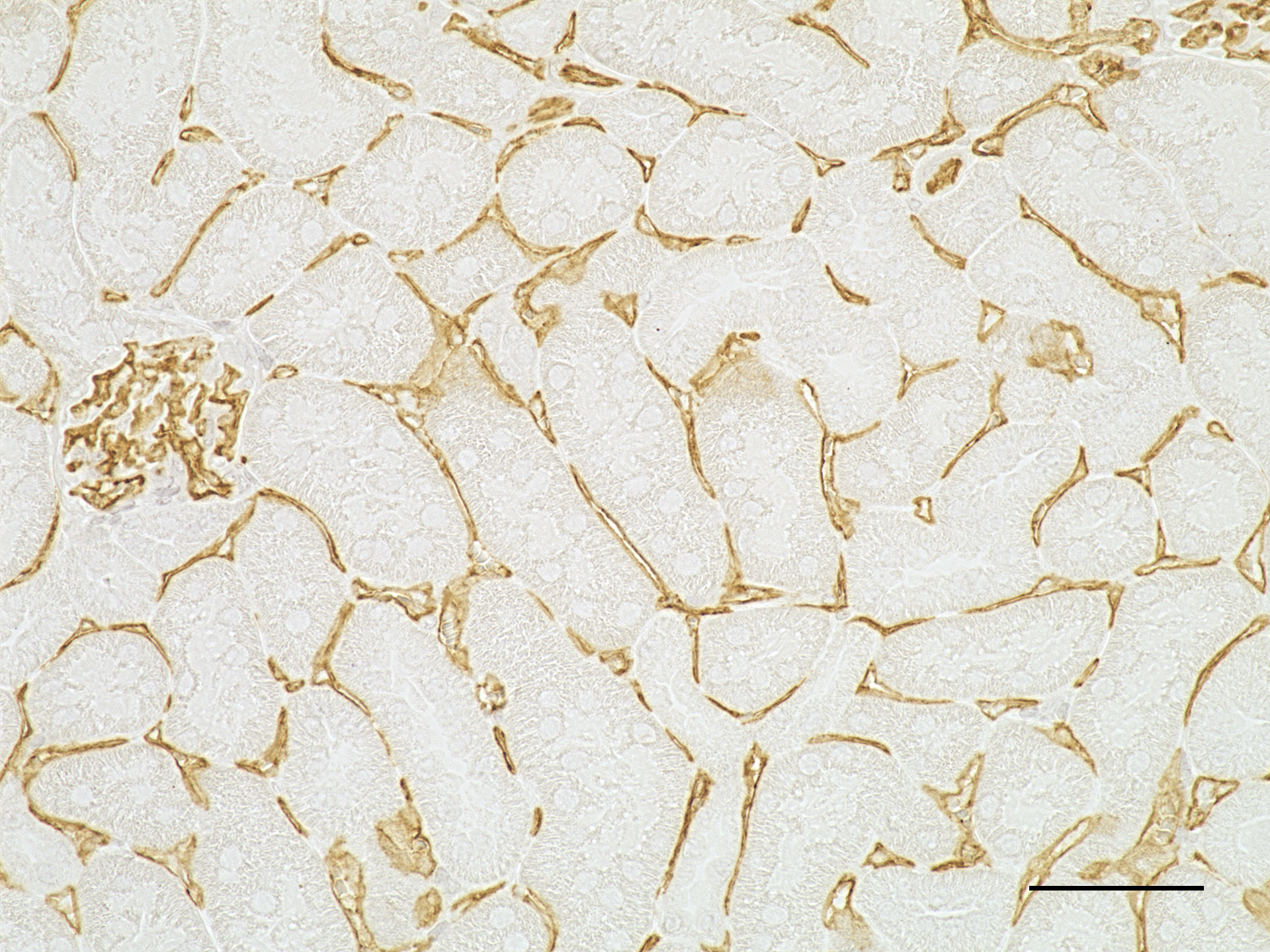

Supplement: Supplementary file 1 [file ijms-25-07683-s001.zip › Supplementary_Material_Microscopy_Images/SupplMat_CD31/WT_sham_058_CD31_3.gif]

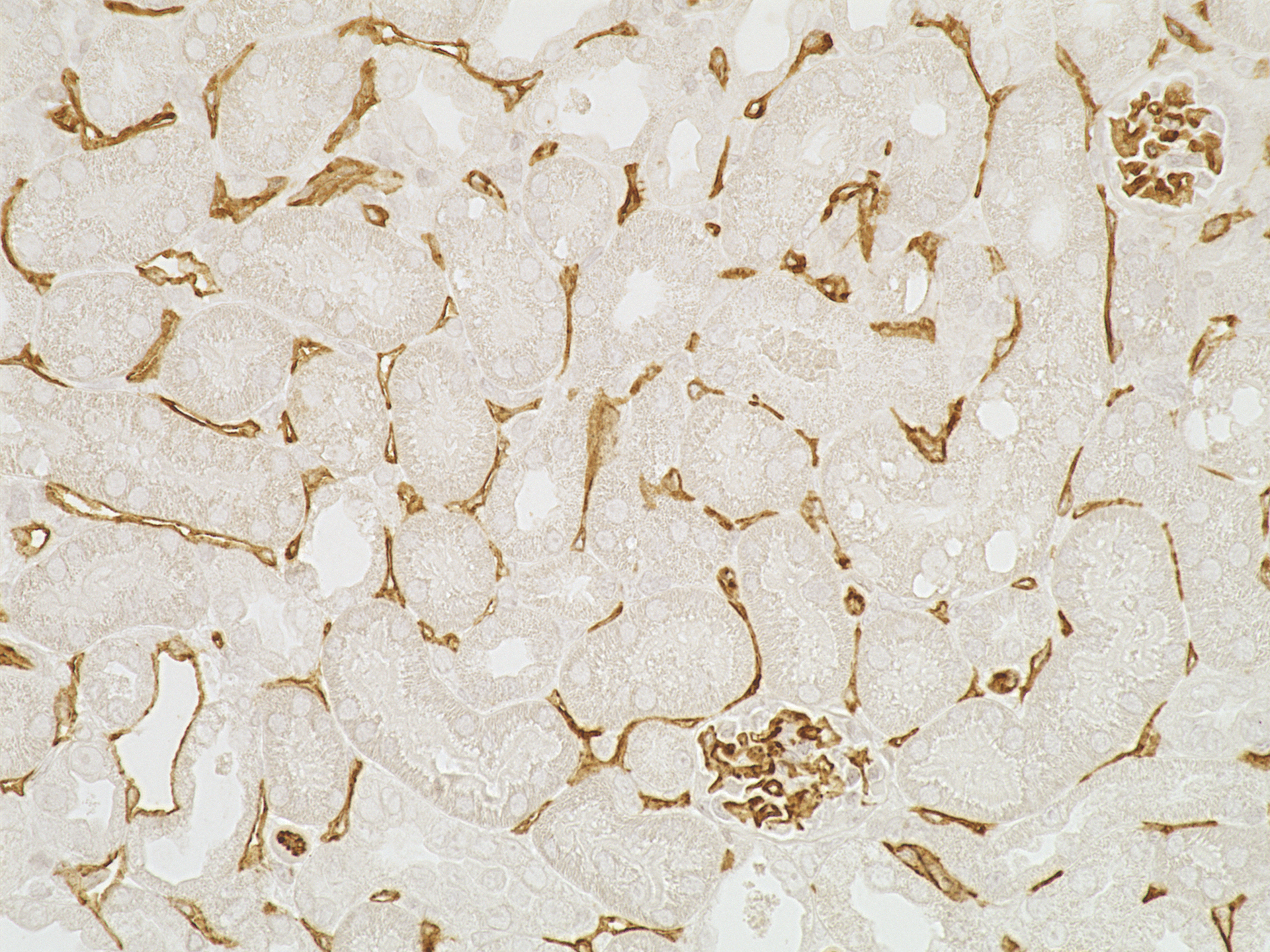

Supplement: Supplementary file 1 [file ijms-25-07683-s001.zip › Supplementary_Material_Microscopy_Images/SupplMat_CD31/WT_Stx_035_CD31_re_6.gif]

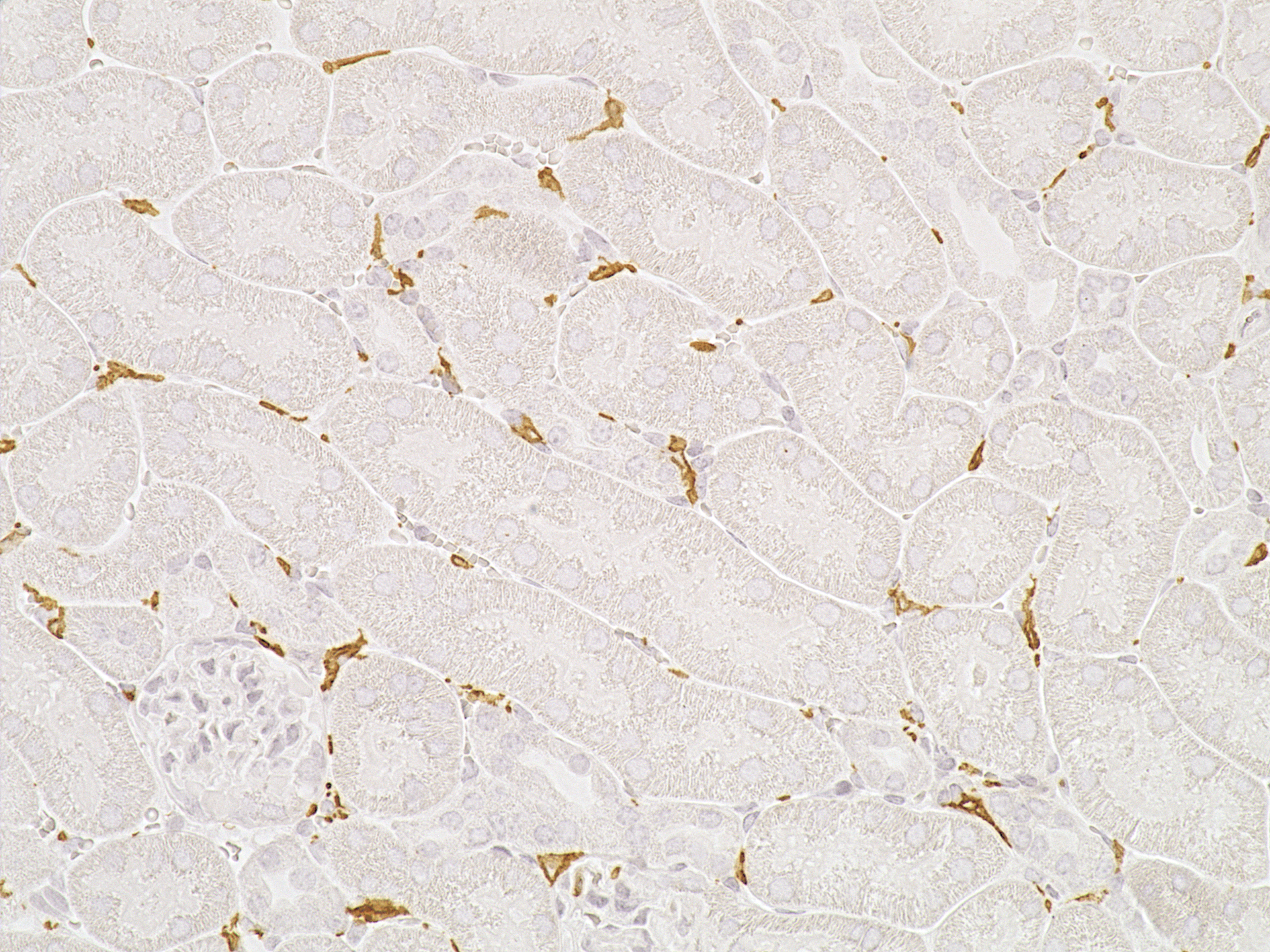

Supplement: Supplementary file 1 [file ijms-25-07683-s001.zip › Supplementary_Material_Microscopy_Images/SupplMat_F4-80/SphK1_sham_008_F4-80_6.gif]

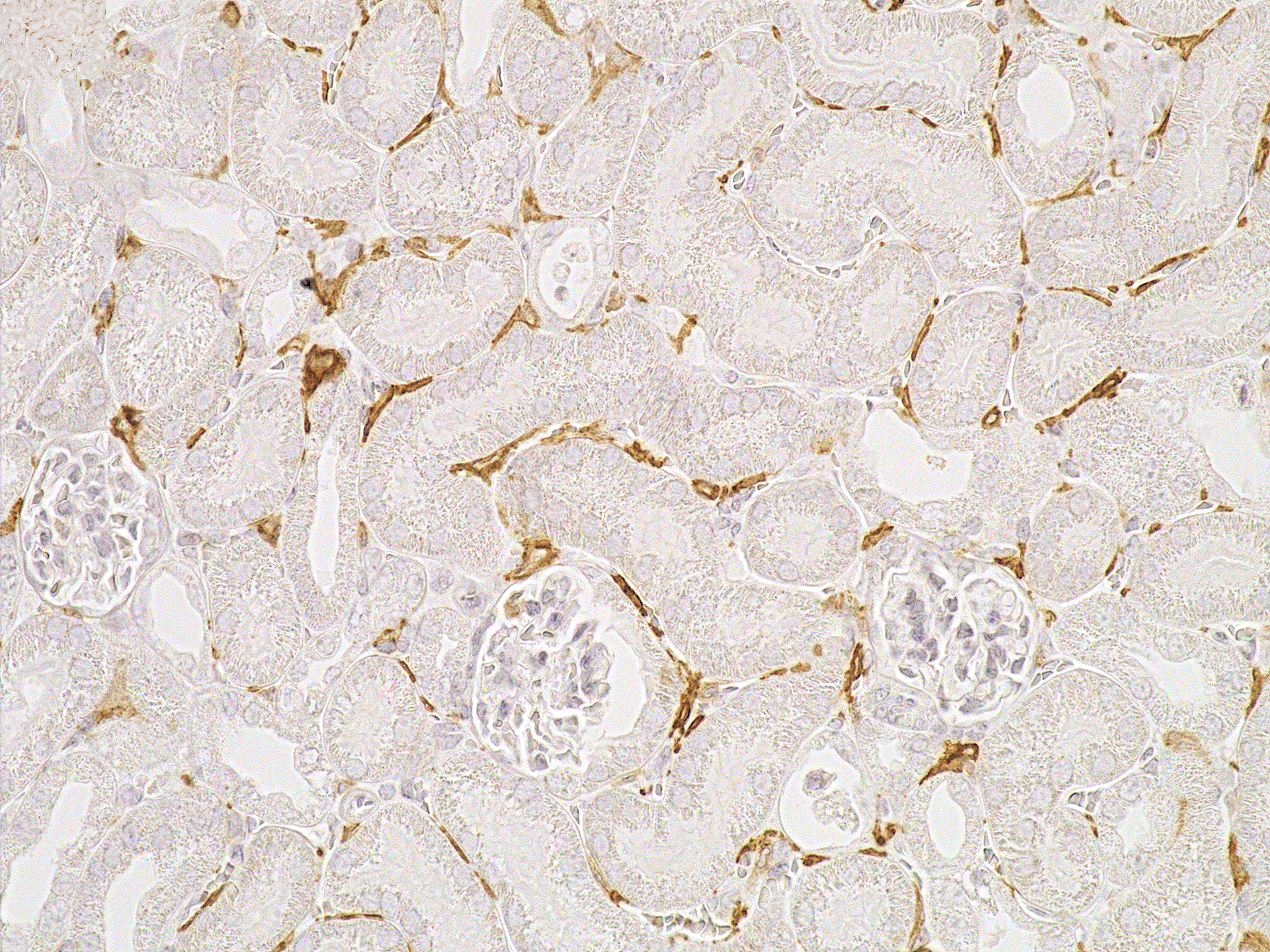

Supplement: Supplementary file 1 [file ijms-25-07683-s001.zip › Supplementary_Material_Microscopy_Images/SupplMat_F4-80/SphK1_Stx_018_F4-80_2.gif]

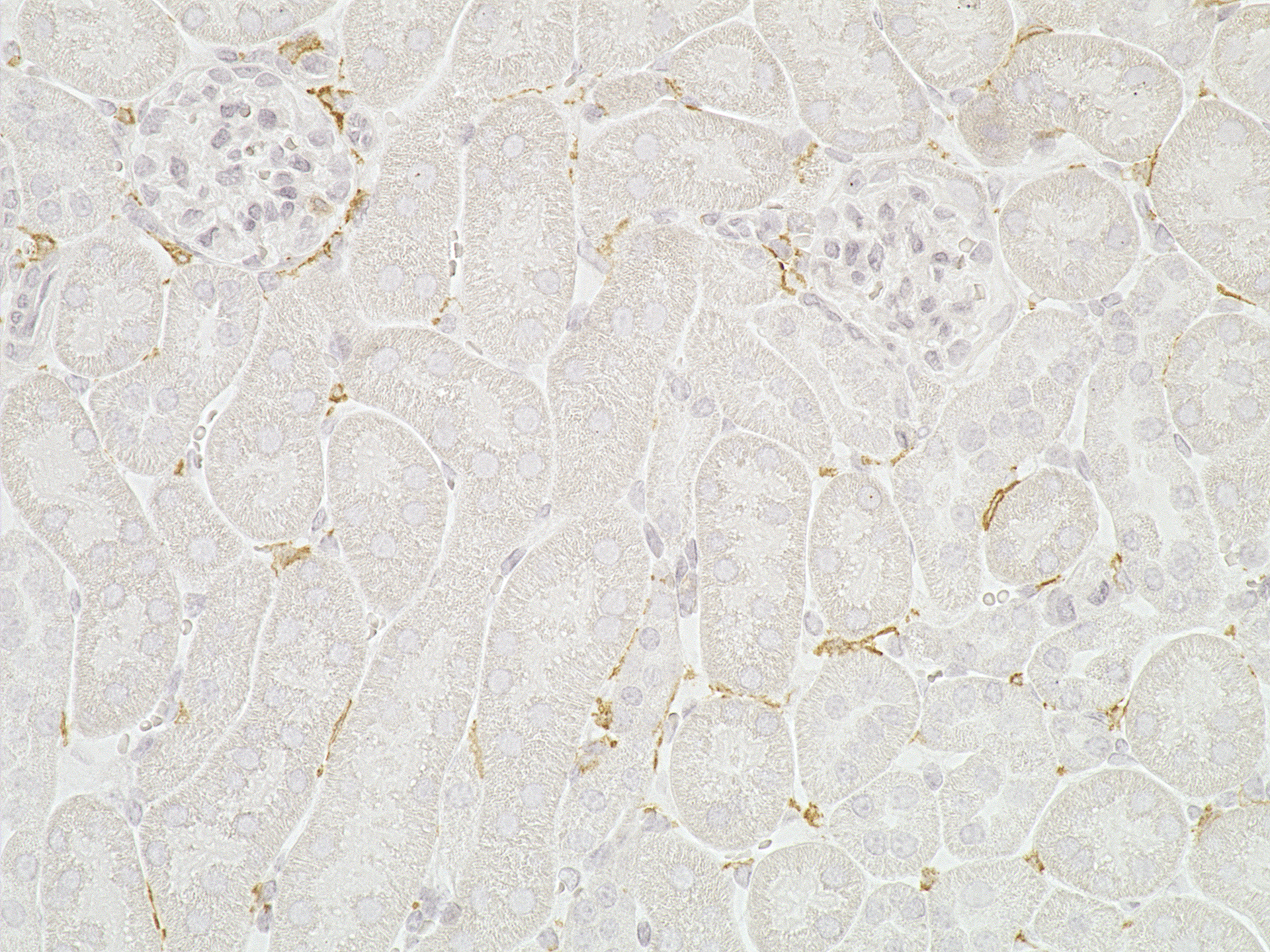

Supplement: Supplementary file 1 [file ijms-25-07683-s001.zip › Supplementary_Material_Microscopy_Images/SupplMat_F4-80/SphK2_sham_019_F4-80_2.gif]

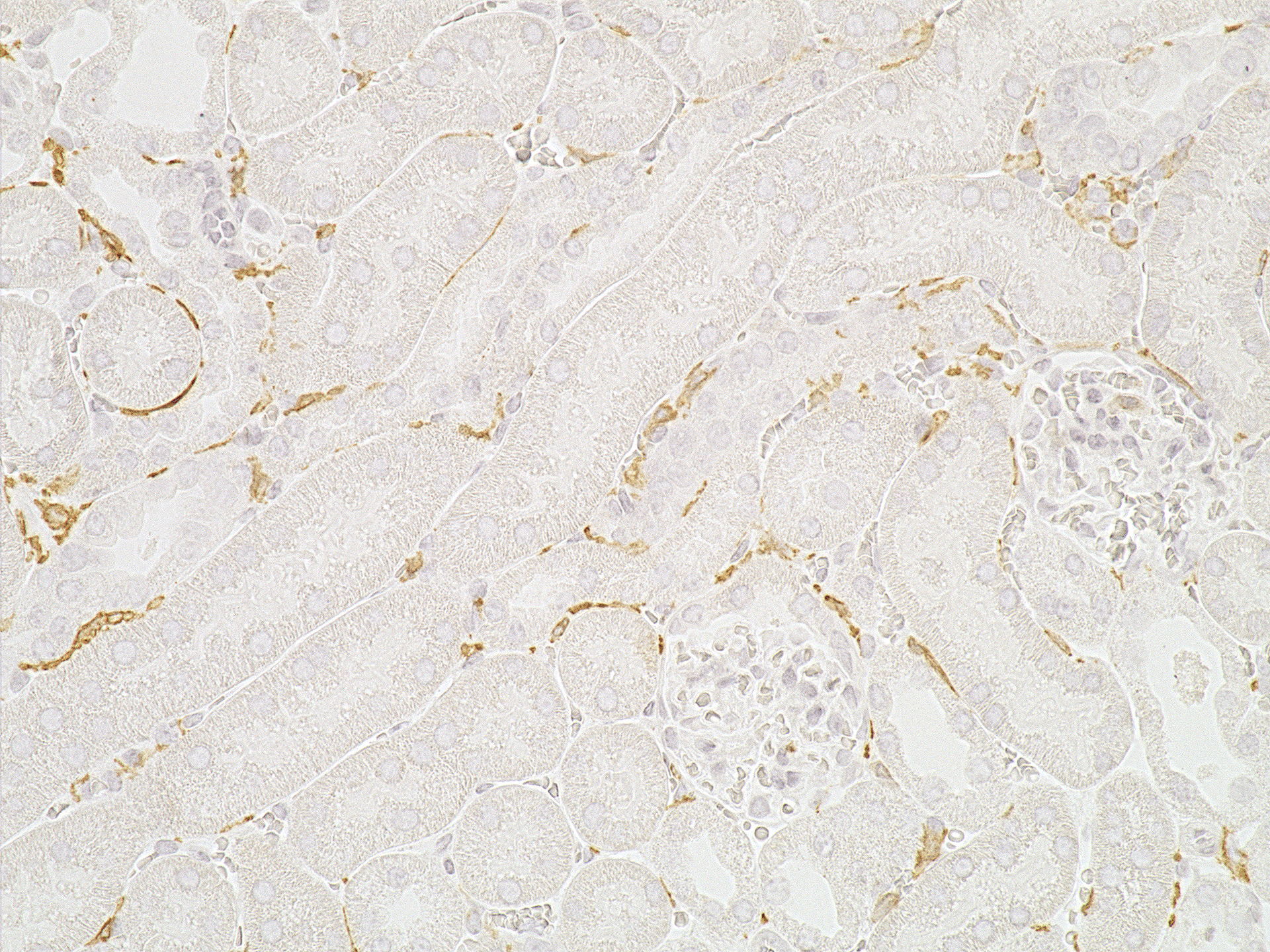

Supplement: Supplementary file 1 [file ijms-25-07683-s001.zip › Supplementary_Material_Microscopy_Images/SupplMat_F4-80/SphK2_Stx_026_F4-80_6.gif]

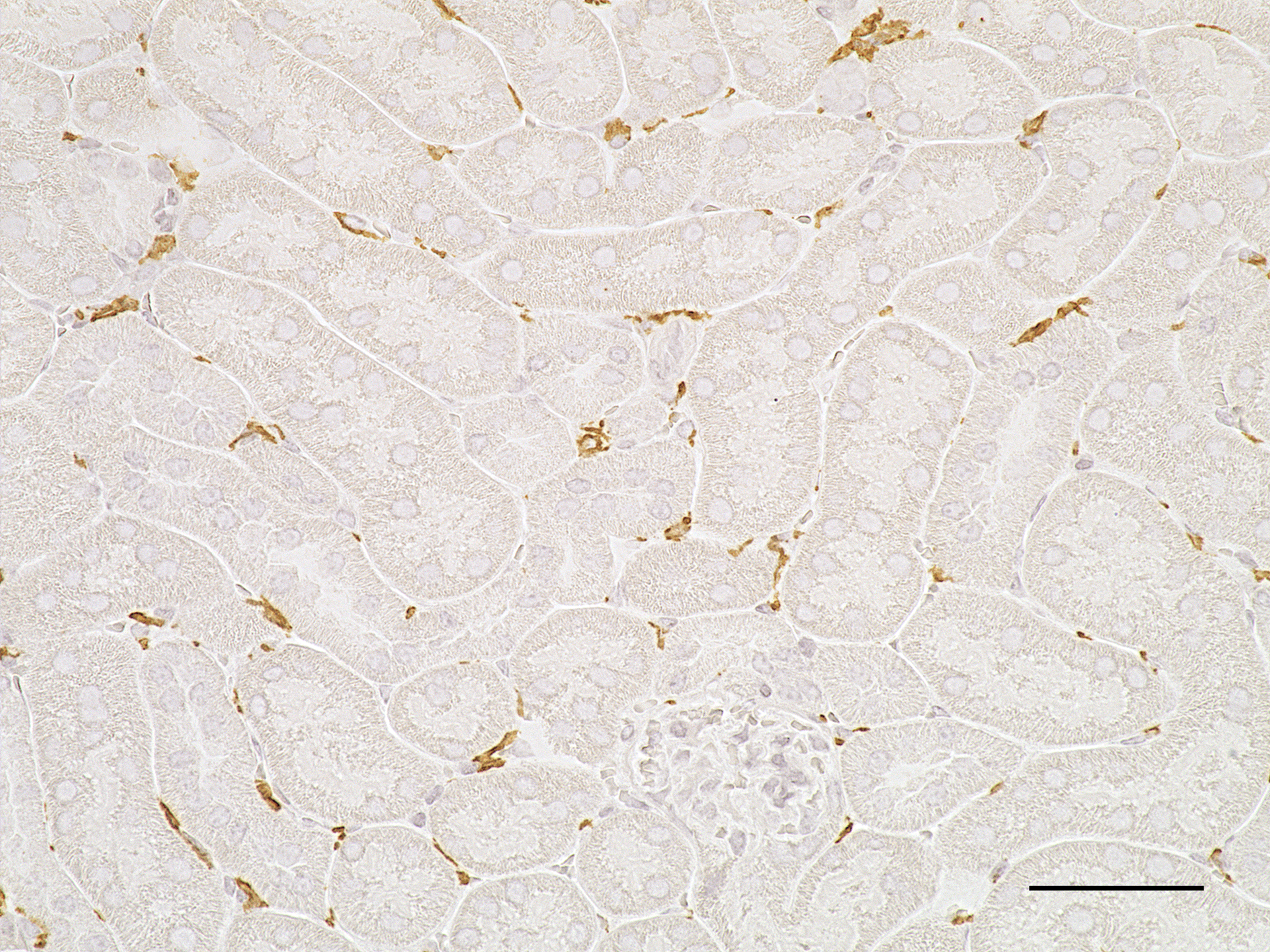

Supplement: Supplementary file 1 [file ijms-25-07683-s001.zip › Supplementary_Material_Microscopy_Images/SupplMat_F4-80/WT_sham_050_F4-80_3.gif]

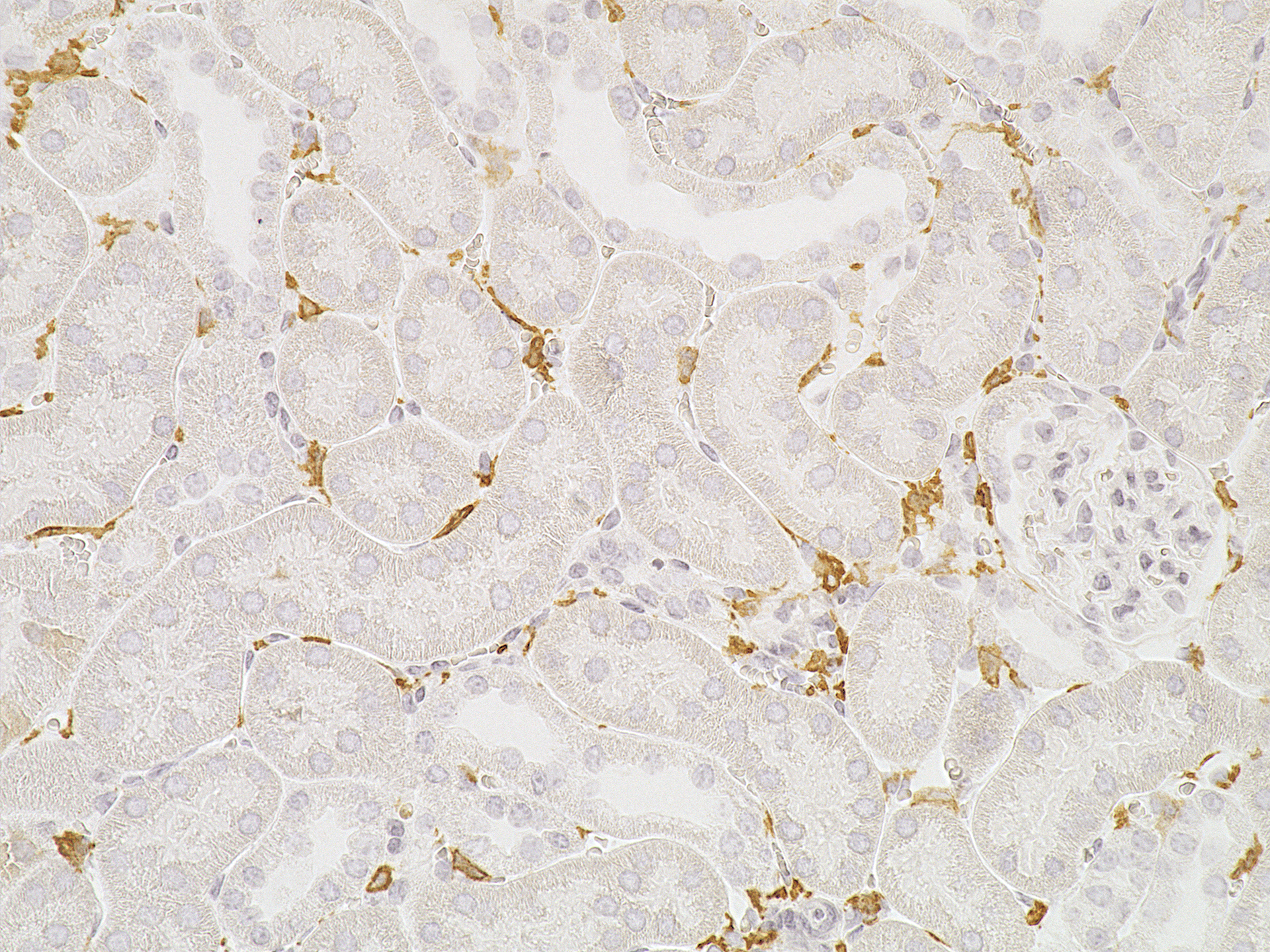

Supplement: Supplementary file 1 [file ijms-25-07683-s001.zip › Supplementary_Material_Microscopy_Images/SupplMat_F4-80/WT_Stx_011_F4-80_2.gif]

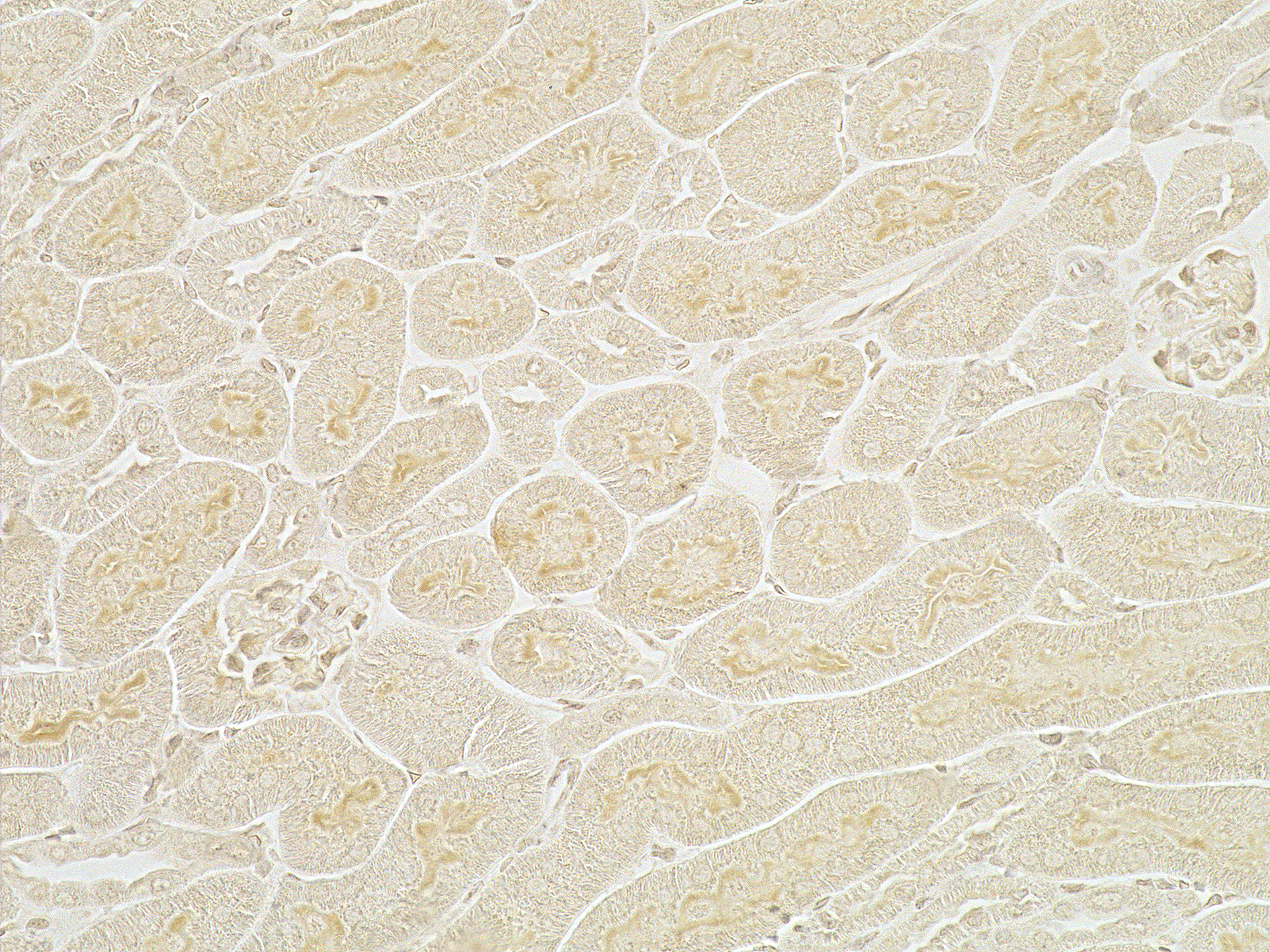

Supplement: Supplementary file 1 [file ijms-25-07683-s001.zip › Supplementary_Material_Microscopy_Images/SupplMat_KIM-1/SphK1_sham_008_KIM-1_11.gif]

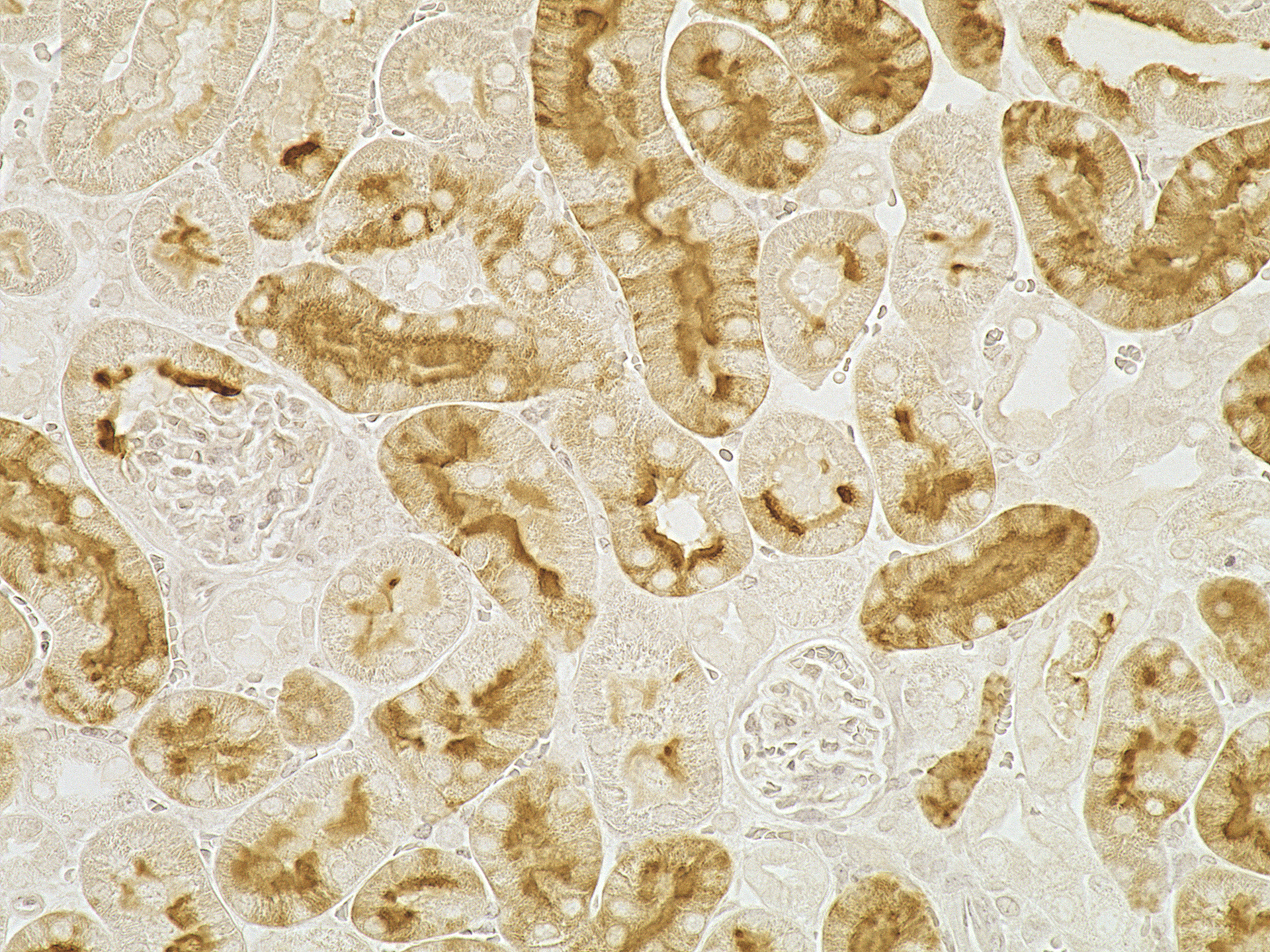

Supplement: Supplementary file 1 [file ijms-25-07683-s001.zip › Supplementary_Material_Microscopy_Images/SupplMat_KIM-1/SphK1_Stx_018_KIM-1_6.gif]

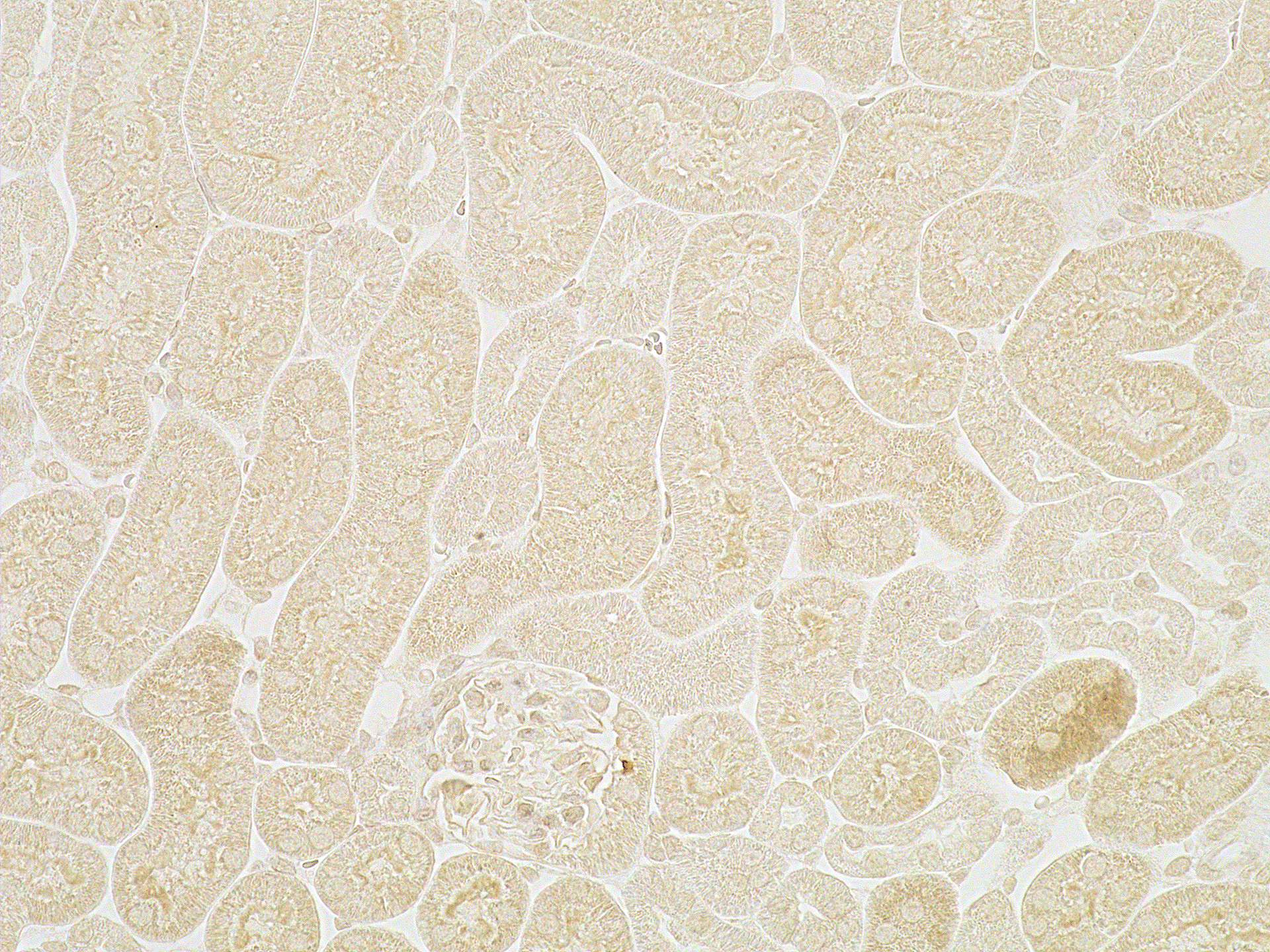

Supplement: Supplementary file 1 [file ijms-25-07683-s001.zip › Supplementary_Material_Microscopy_Images/SupplMat_KIM-1/SphK2_sham_027_KIM-1_6.gif]

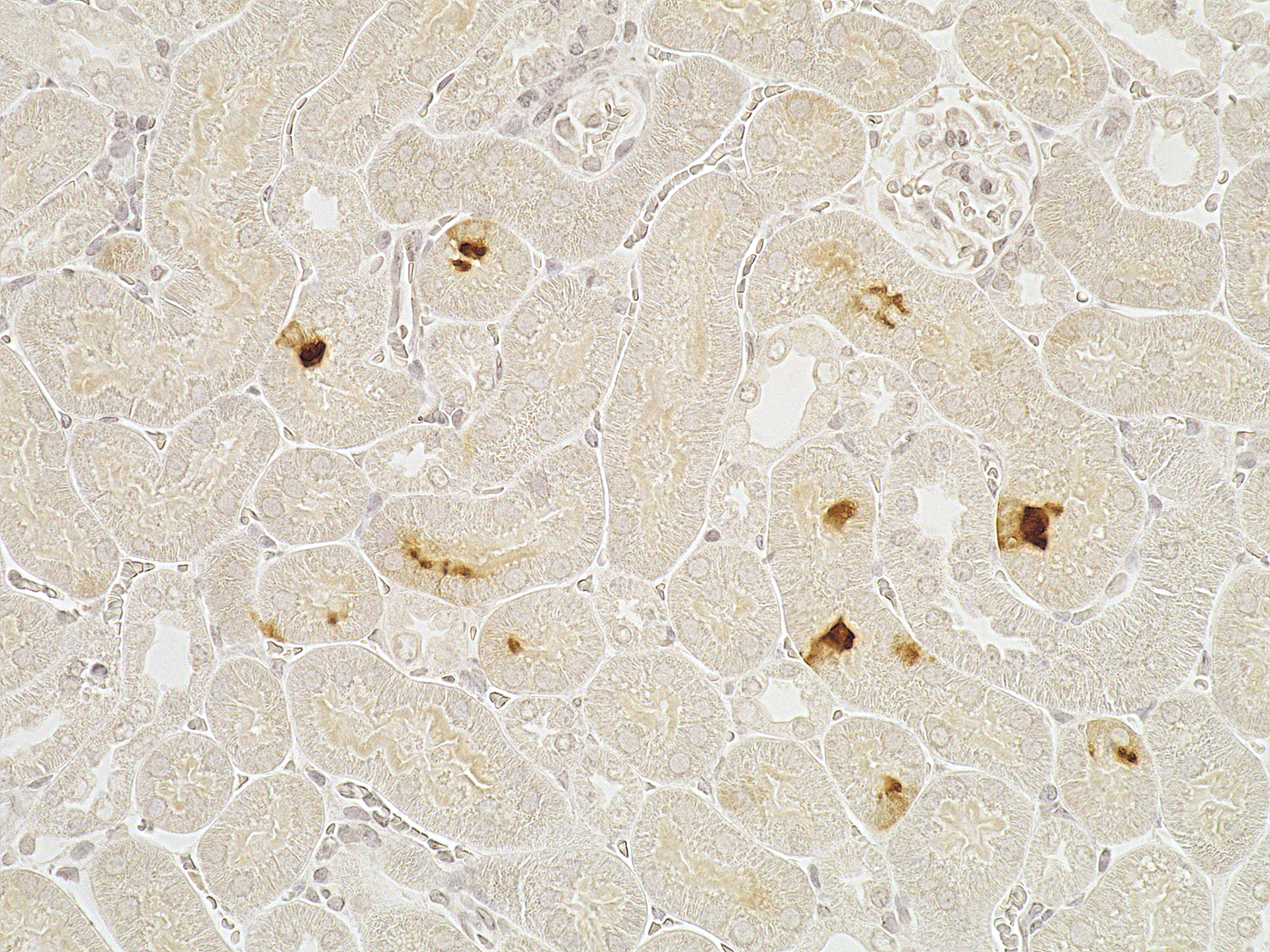

Supplement: Supplementary file 1 [file ijms-25-07683-s001.zip › Supplementary_Material_Microscopy_Images/SupplMat_KIM-1/SphK2_Stx_028_KIM-1_8.gif]

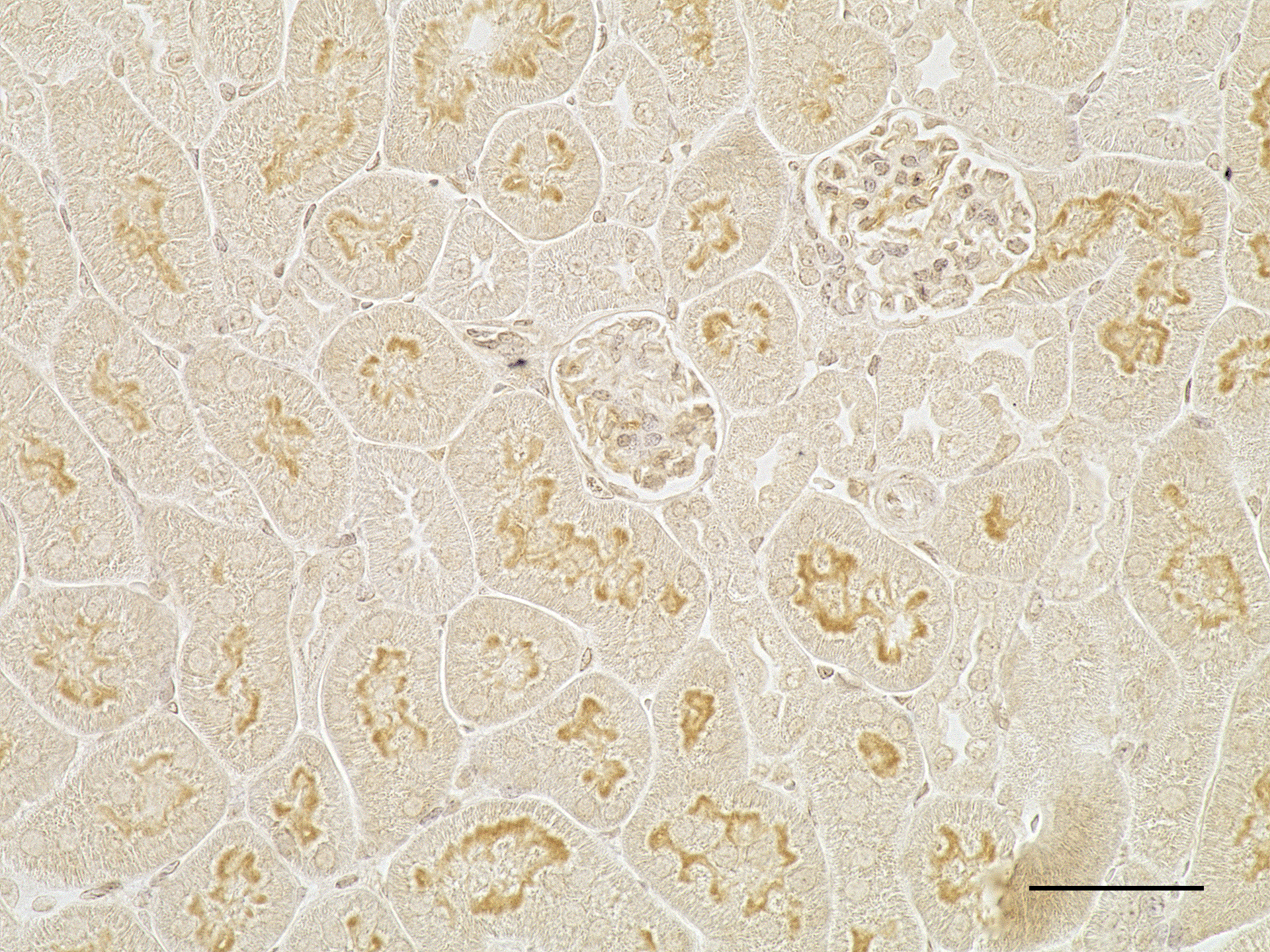

Supplement: Supplementary file 1 [file ijms-25-07683-s001.zip › Supplementary_Material_Microscopy_Images/SupplMat_KIM-1/WT_sham_058_KIM-1_5.gif]

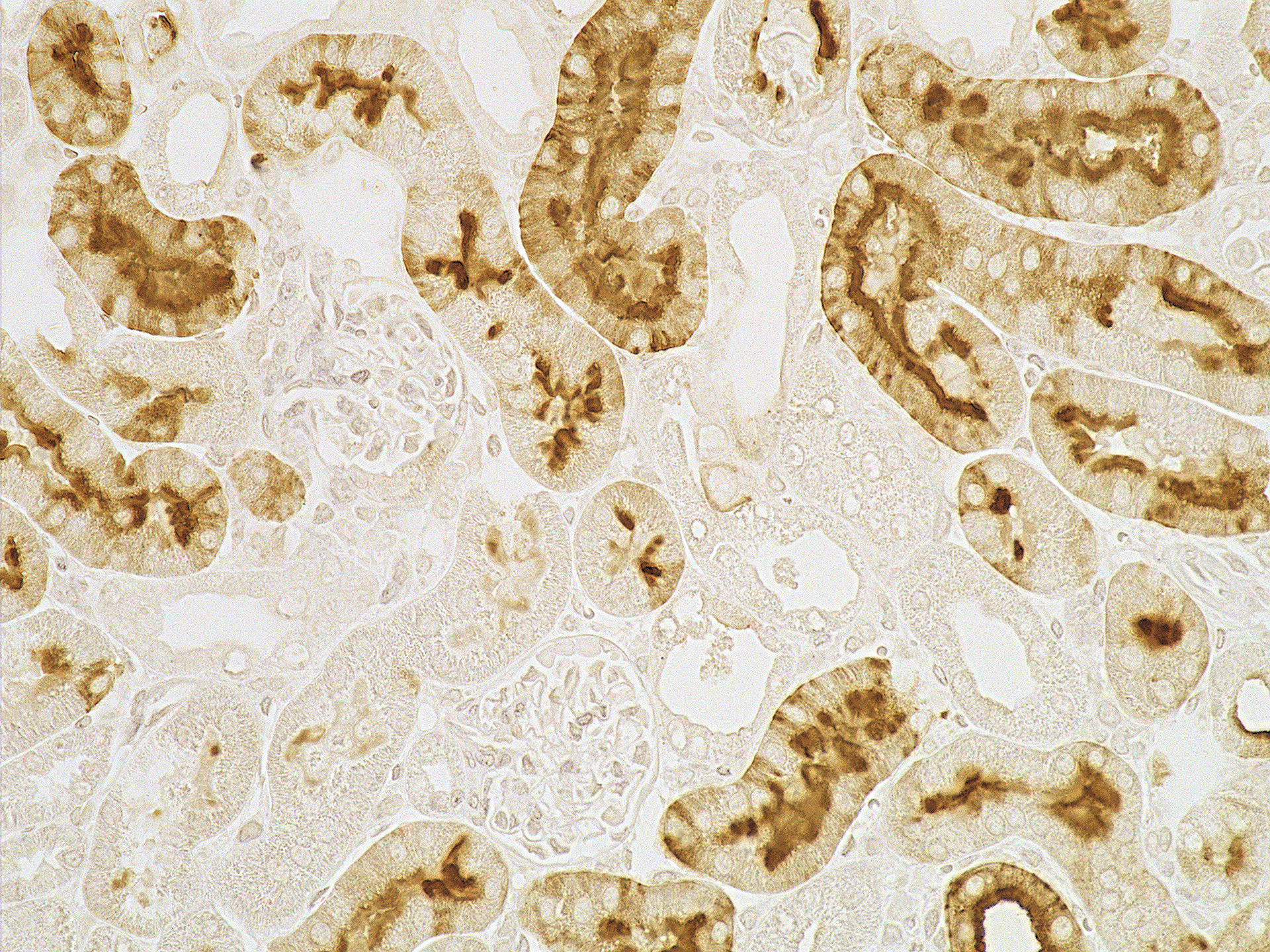

Supplement: Supplementary file 1 [file ijms-25-07683-s001.zip › Supplementary_Material_Microscopy_Images/SupplMat_KIM-1/WT_Stx_017_PAS_TM_6.gif]

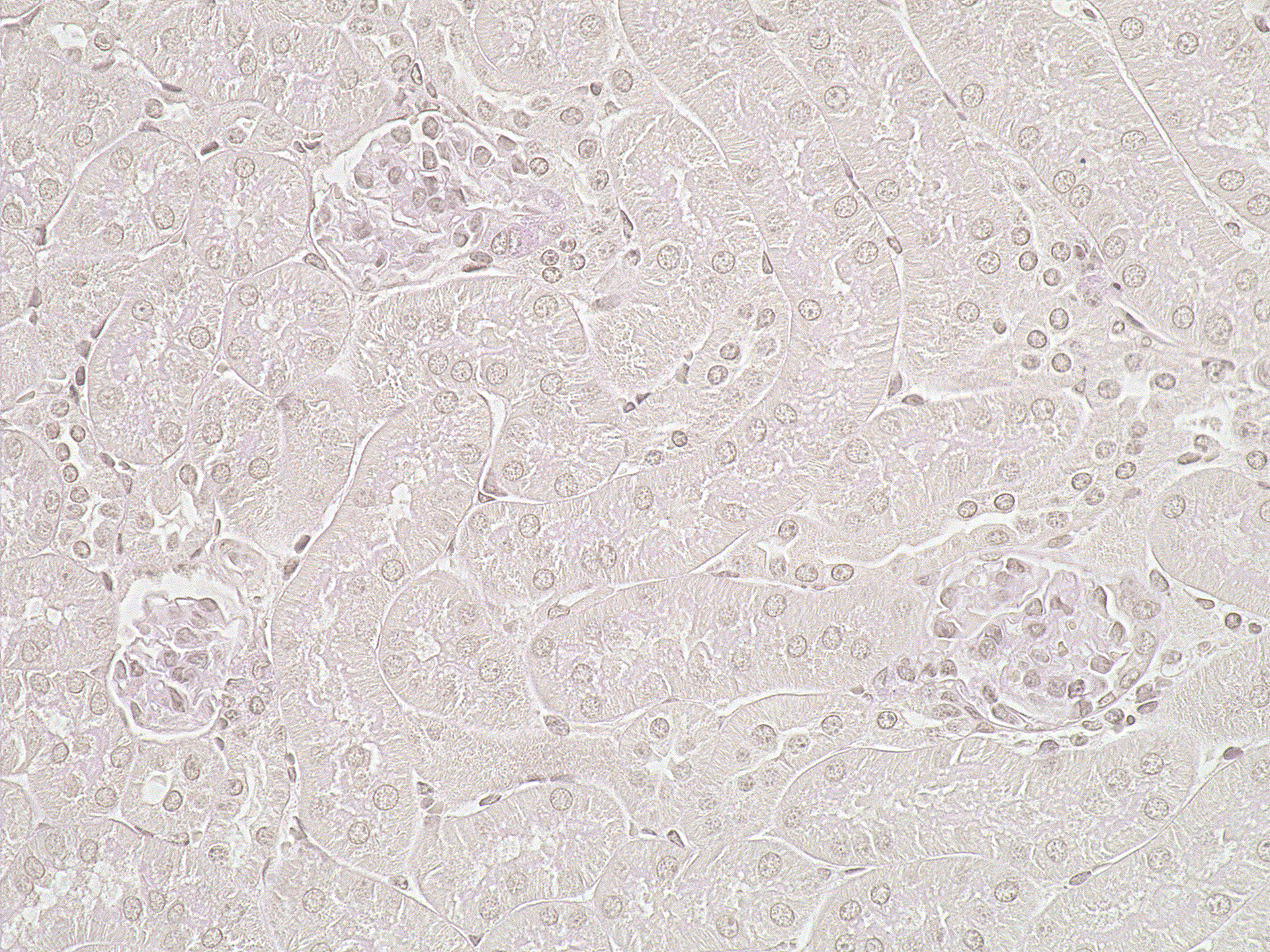

Supplement: Supplementary file 1 [file ijms-25-07683-s001.zip › Supplementary_Material_Microscopy_Images/SupplMat_PAS/SphK1_sham_025_PAS_6.gif]

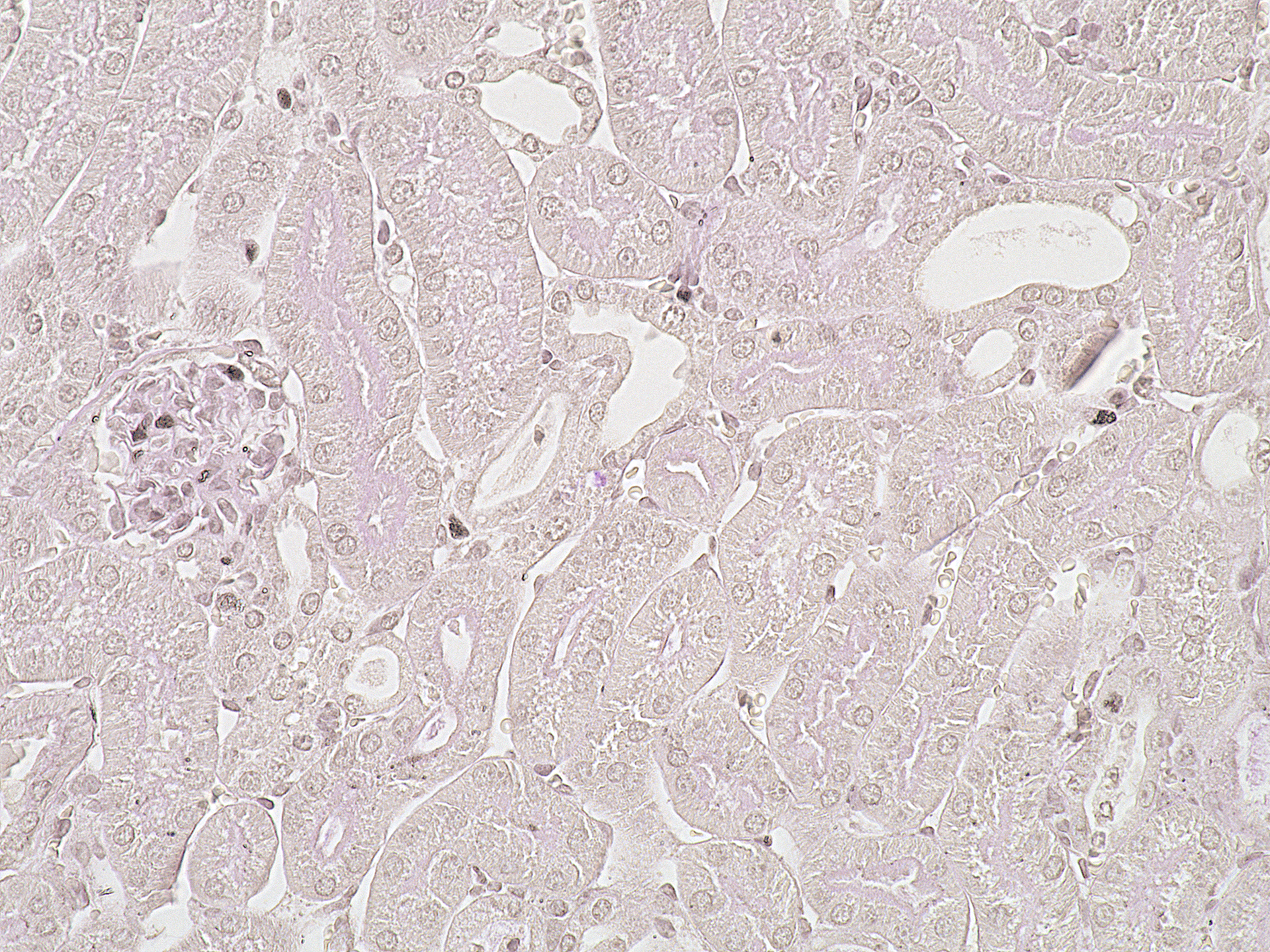

Supplement: Supplementary file 1 [file ijms-25-07683-s001.zip › Supplementary_Material_Microscopy_Images/SupplMat_PAS/SphK1_Stx_018_PAS_6.gif]

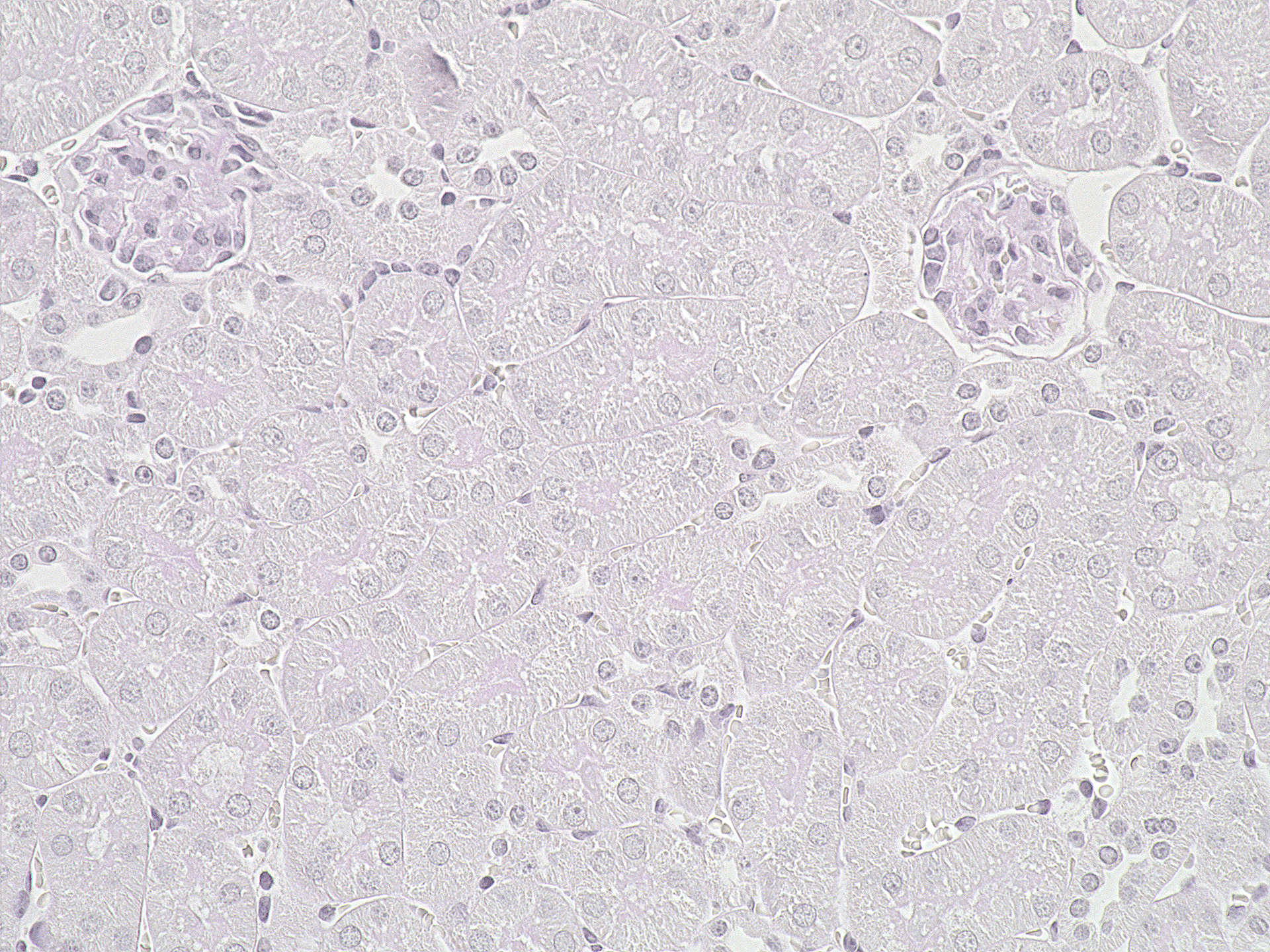

Supplement: Supplementary file 1 [file ijms-25-07683-s001.zip › Supplementary_Material_Microscopy_Images/SupplMat_PAS/SphK2_sham_019_PAS_6.gif]

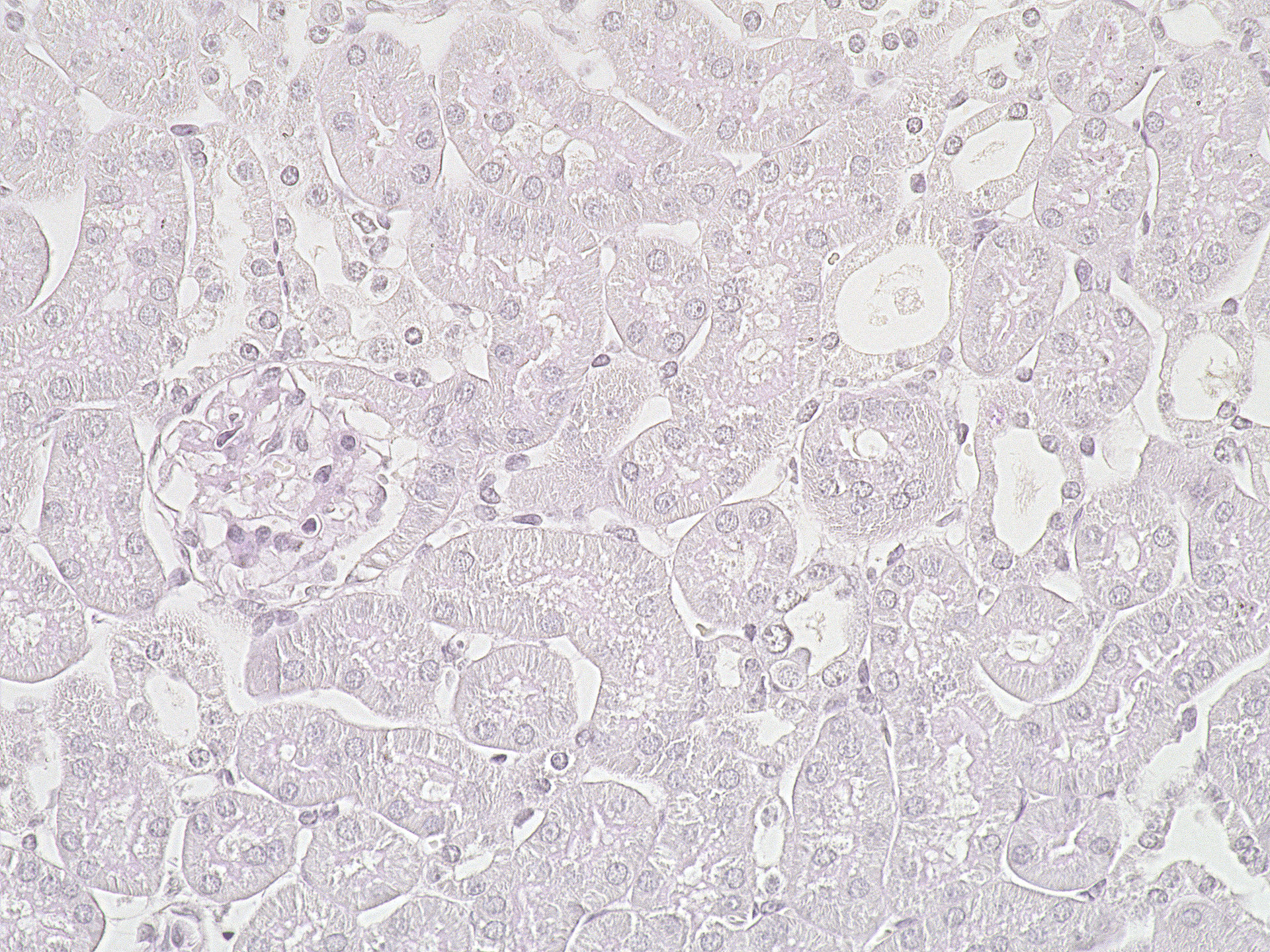

Supplement: Supplementary file 1 [file ijms-25-07683-s001.zip › Supplementary_Material_Microscopy_Images/SupplMat_PAS/SphK2_Stx_011_PAS_4.gif]

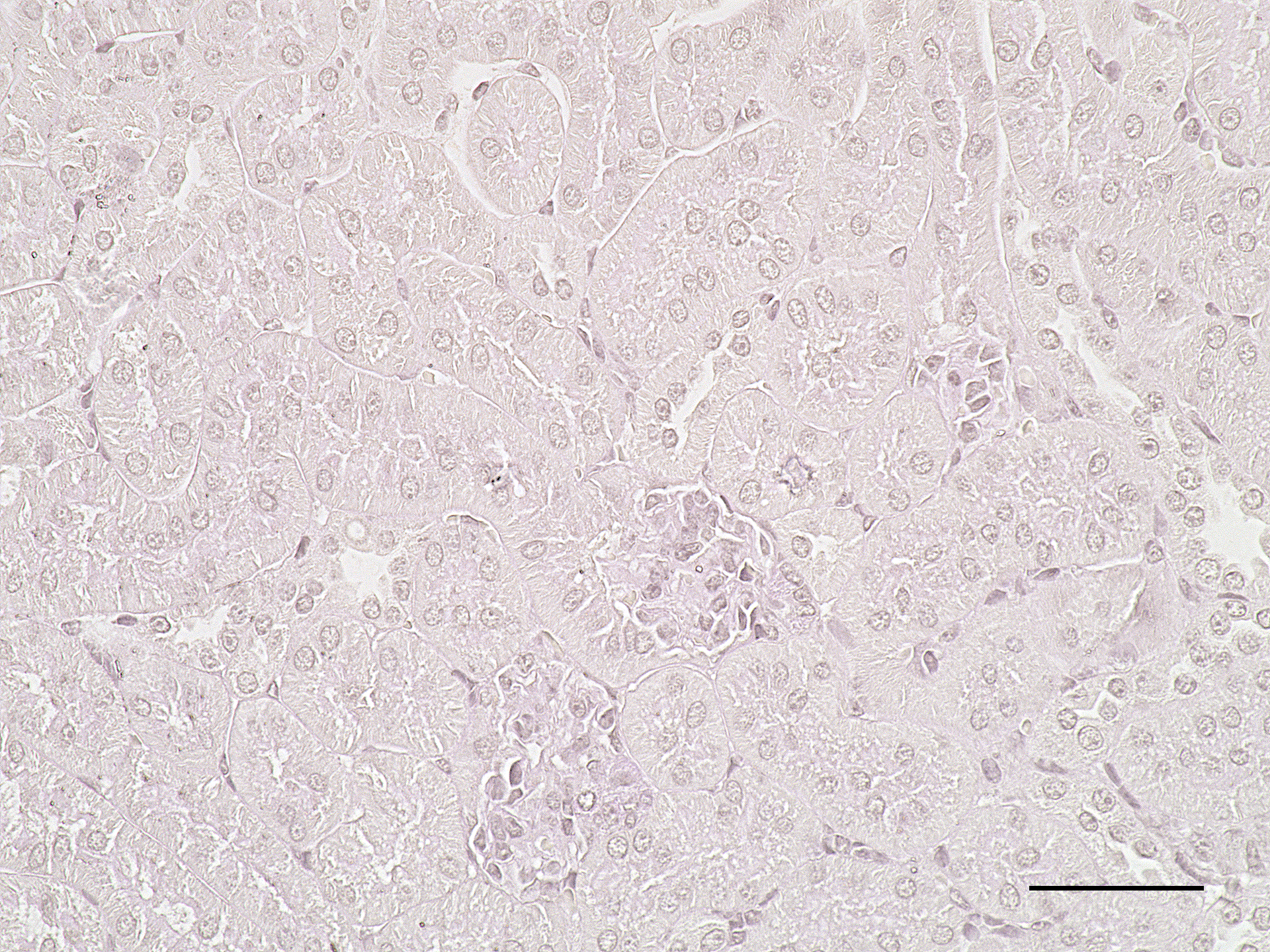

Supplement: Supplementary file 1 [file ijms-25-07683-s001.zip › Supplementary_Material_Microscopy_Images/SupplMat_PAS/WT_sham_027_PAS_7.gif]

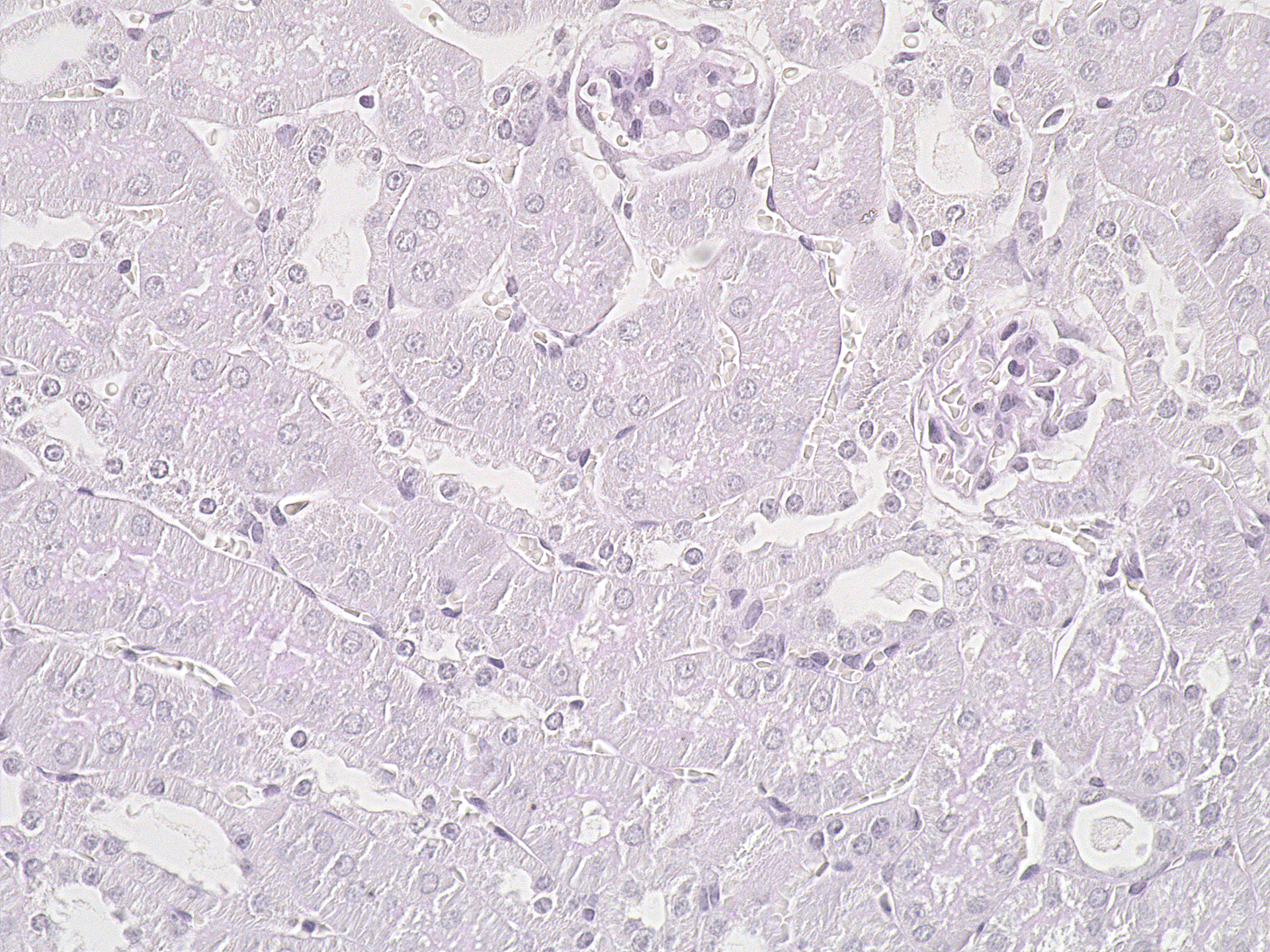

Supplement: Supplementary file 1 [file ijms-25-07683-s001.zip › Supplementary_Material_Microscopy_Images/SupplMat_PAS/WT_Stx_054_PAS_6.gif]
